# Selective Restoration of *Pomc* Expression in Glutamatergic POMC Neurons: Evidence for a Dynamic Hypothalamic Neurotransmitter Network

**DOI:** 10.1523/ENEURO.0400-18.2019

**Published:** 2019-04-01

**Authors:** Graham L. Jones, Gábor Wittmann, Eva B. Yokosawa, Hui Yu, Aaron J. Mercer, Ronald M. Lechan, Malcolm J. Low

**Affiliations:** 1 Neuroscience Graduate Program; 2 Department of Molecular and Integrative Physiology, University of Michigan Medical School, Ann Arbor, MI 48105; 3 Department of Medicine, Division of Endocrinology, Diabetes and Metabolism, Tufts Medical Center, Boston, MA 02111

**Keywords:** GABA, glutamate, neurotransmitter flexibility, obesity, POMC

## Abstract

Hypothalamic POMC deficiency leads to obesity and metabolic deficiencies, largely due to the loss of melanocortin peptides. However, POMC neurons in the arcuate nucleus (ARC) are comprised of glutamatergic and GABAergic subpopulations. The developmental program, relative proportion and function of these two subpopulations are unresolved. To test whether glutamatergic POMC neurons serve a distinct role in maintaining energy homeostasis, we activated *Pomc* expression Cre- dependently in *Vglut2*-expressing neurons of mice with conditionally silenced *Pomc* alleles. The *Vglut2*-*Pomc* restored mice had normal ARC *Pomc* mRNA levels, POMC immunoreactivity, as well as body weight and body composition at age 12 weeks. Unexpectedly, the cumulative total of *Vglut2^+^* glutamatergic- and *Gad67^+^ GABAergic*-*Pomc* neurons detected by *in situ* hybridization (ISH) exceeded 100% in both *Vglut2*- *Pomc* restored and control mice, indicating that a subpopulation of *Pomc* neurons must express both neuronal markers. Consistent with this hypothesis, triple ISH of C57BL/6J hypothalami revealed that 35% of ARC *Pomc* neurons were selectively *Gad67* ^+^, 21% were selectively *Vglut2* ^+^, and 38% expressed both *Gad67* and *Vglut2*. The single *Gad67* ^+^ and *Vglut2* ^+^ *Pomc* neurons were most prevalent in the rostral ARC, while the *Vglut2/Gad67* ^+^ dual-phenotype cells predominated in the caudal ARC. A lineage trace using Ai9-tdTomato reporter mice to label fluorescently all *Vglut2*-expressing neurons showed equal numbers of tdTomato^+^ and tdTomato^-^ POMC immunoreactive neurons. Together, these data suggest that POMC neurons exhibit developmental plasticity in their expression of glutamatergic and GABAergic markers, enabling re-establishment of normal energy homeostasis in the *Vglut2*-*Pomc* restored mice.

## Significance Statement

Melanocortin peptides secreted from hypothalamic POMC neurons are anorexigenic and play a critical role in preventing obesity. However, POMC neurons are heterogeneous in their synaptic release of the neurotransmitters glutamate and GABA. We used a conditional gene expression approach to test the hypothesis that these two subsets of POMC neurons influence distinct neural circuits controlling energy homeostasis. Unexpectedly, we found that *Vglut2*-IRES-Cre dependent *Pomc* restoration on a hypothalamic *Pomc* null background was sufficient to prevent obesity. A series of double-label and triple-label *in situ* hybridization (ISH) experiments showed that 38% of POMC neurons express both *Slc17a6* and *Gad67*, markers of glutamatergic and GABAergic neurons, respectively. Together, these data suggest a previously unreported developmental plasticity in the neurotransmitter phenotype of POMC neurons.

## Introduction

POMC-derived peptides are critical in maintaining energy balance and body composition, as well as in regulating feeding behavior. Neuronal *Pomc* deficiency leads to morbid obesity, hyperphagia, hypolocomotion, and metabolic abnormalities (Bumaschny et al., 2012; Lam et al., 2015b; Chhabra et al., 2016a,b). It was identified previously that POMC neurons are comprised of both GABAergic and glutamatergic cells (Hentges et al., 2004, 2009; Mercer et al., 2013). However, little is known about the functional impact or genetic programs of these neuronal subclasses. Furthermore, there is not a consensus on the relative proportions of each POMC neuron subtype, due to differences in methodologies used to classify the cells (Hentges et al., 2009; Vong et al., 2011; Dicken et al., 2012; Wittmann et al., 2013; Dennison et al., 2016).

Many studies have revealed plasticity in neurotransmitter identity or in neurotransmitter co-release. These phenomena are evident during development or in response to environmental stimuli, and span diverse cell groups and neurotransmitter types (Walker et al., 2001; Gutiérrez, 2002, 2003; Gutiérrez et al., 2003; Kao et al., 2004; Ottem et al., 2004; Gillespie et al., 2005; Gómez-Lira et al., 2005; Zander et al., 2010; Mestikawy et al., 2011; Dulcis et al., 2013; Root et al., 2014; Spitzer, 2015, 2017; Meng et al., 2018). Work by Dennison et al. (2016) uncovered a postnatal shift in the proportions of *Vglut2*^+^ and *Gad67*^+^ POMC neurons, where the *Vglut2-Pomc* overlap was the highest (∼40%) immediately after birth, but was reduced by 4-fold (∼10%) when the animals had matured to eight weeks old. The opposite temporal pattern was observed with *Gad67-Pomc* overlap. However, the mechanism of this shift in proportionality is unclear. One possibility is that POMC neurons are glutamatergic early in hypothalamic development and then transdifferentiate to a GABAergic phenotype in postnatal life. Alternatively, there may be a selective increase in the absolute number of de novo GABAergic POMC neurons that arise postnatally. There are also reports of some POMC neurons expressing both *Vglut2*^+^ and *Gad67*^+^ (Jarvie and Hentges, 2012), indicating the possibility of a shift between neurotransmitter phenotypes or the potential that a subset of POMC neurons can synaptically release both glutamate and GABA. Recent work from Stincic et al. (2018) also indicates that POMC neurons can locally regulate the function of neuropeptide Y/agouti-related peptide neurons in the arcuate nucleus (ARC) via glutamatergic and β-endorphin input, expanding our functional understanding of melanocortin circuitry. Additionally, there are data indicating a partial dissociation between peptidergic and fast neurotransmitter synaptic terminals from POMC neuron projections at sites throughout the brain (Mercer et al., 2014), further complicating the interpretation of the specific functions of these neurons.

This study was conceived initially to test the hypothesis that glutamatergic and GABAergic POMC neurons serve distinct and dissociable roles in overall POMC neuron function related to the maintenance of energy homeostasis. We chose to investigate the impact of selectively restoring *Pomc* function in the developing hypothalamus from a conditionally silent allele (FneoΔ2 mice) using a *Vglut2*-IRES-Cre knock-in mouse model (Vong et al., 2011) and then determine how restoration of *Pomc* expression only in the glutamatergic subpopulation of POMC neurons shapes hypothalamic POMC neural circuitry and impacts energy balance in the obesity-destined mice. Additionally, we sought to capture the overlap between glutamate-associated neurons and hypothalamic POMC expression. We also used *Vglut2*-Cre-driven reporter expression to perform a lineage trace of all cells that have expressed *Vglut2* at some point in their existence to compare with POMC immunoreactivity. Finally, triple-label *in situ* hybridization (ISH) was performed on wild-type tissue to establish the degree of overlap between *Pomc*, *Vglut2*, and *Gad67* gene expression in adult mice.

## Materials and Methods

### Animal care

All animal procedures were performed in accordance with the University of Michigan IACUC regulations. Mice were housed under a 12/12 h light/dark photoperiod at constant temperature of 22°C in ventilated cages with *ad libitum* access to water and chow (5L0D; LabDiet containing 28.5 kcal% protein, 13.5 kcal% fat, and 58.0 kcal% carbohydrates).

### Mouse strains and breeding strategy

Ai9 tdTomato reporter mice (Allen Institute, The Jackson Laboratory; *Gt(ROSA)26Sor^tm9(CAG-tdTomato)Hze^*) were crossed to *Vglut2*-ires-Cre/^+^ mice (The Jackson Laboratory; *Slc17a6^tm2(cre)Lowl^*/J; Vong et al., 2011) to generate *Vglut2*-tdTomato compound mice for a developmental lineage trace of all neurons that have expressed the gene encoding the vesicular glutamate transporter Vglut2 at some point in their existence. Male (M) and female (F) mice were used in all experiments.

*ArcPomc^+/-^* (ARC specific Cre-reversible *Pomc* KO or FneoΔ2) mice (Bumaschny et al., 2012; Lam et al., 2015a; Chhabra et al., 2016a,b) were crossed to *Vglut2* ^Cre/+^ mice to obtain compound heterozygous *Vglut2*^Cre/+^; *arcPomc*^+/-^ mice. Those mice were back crossed to *arcPomc* ^+/-^ mice to yield the control and experimental groups for POMC restoration and ISH studies. These three groups were: *Vglut2* ^Cre/+^; *Pomc* ^+/+^ (control), *Vglut2*^+/+^; *arcPomc* ^-/-^ (FNΔ2) and *Vglut2* ^Cre/+^; *arcPomc* ^-/-^ (restored). FNΔ2 animals have a floxed-neomycin cassette inserted between neural *Pomc* enhancer 1 (nPE1) and the deleted neural *Pomc* enhancer 2 (ΔnPE2) locus, which prevents the transcription of *Pomc* in neurons, while leaving pituitary transcription intact. After Cre-mediated excision of the floxed-neomycin cassette, neuronal *Pomc* transcription is restored from the functional nPE1 enhancer.

### Growth curves, body composition, and tissue collection

Mice were weighed weekly from ages 3–12 weeks. Body composition was assessed by nuclear magnetic resonance (NMR; Minispec LF90 II, Bruker Optics) at age 12 weeks. Following NMR, a cohort of animals was killed by decapitation; gonadal and inguinal fat pads were collected and weighed, and bilateral 2-mm^3^ tissue blocks were collected from the medial-basal hypothalamus (coordinates from bregma; A-P: –1 to –3 mm, M-L: ±0 to 1 mm, and D-V: 0 to 1 mm from ventral surface) and the dorsal striatum (coordinates from bregma; A-P: +1 to –1 mm, M-L: ±1 to 2 mm, and D-V: –2.5 to –3.5 mm from dorsal surface) for use in genomic DNA PCR and qRT-PCR. The brains used in the ISH studies were collected at age 9–13 weeks, and fresh tissue was flash frozen using isopentane (2-methyl butane) cooled on dry-ice. Tissue used for immunohistochemistry (IHC) was collected from 12- to 13-week-old mice, anesthetized with an overdose of 2% tribromoethanol (Avertin; 400 mg/kg, i.p.) and perfused transcardially with PBS (pH 7.4), followed by 4% paraformaldehyde (PFA; Sigma-Aldrich; catalog #158127) dissolved in PBS (pH 7.4). Brains were post-fixed overnight in 4% PFA at 4°C, and then cryoprotected with 30% sucrose (ThermoFisher Scientific; catalog #BP220) in PBS (pH 7.4).

### PCR verification of Cre-mediated genomic DNA recombination in restored mice

Genomic DNA samples were extracted from one of the bilateral 2-mm^3^ blocks of fresh brain tissue described above. The samples were then analyzed by PCR using primers designed to detect the presence of the floxed neomycin cassette and the recombined DNA sequence following Cre-mediated excision of the neo cassette (forward1: TACTTGGGCCTCAGGGTACTGAAA, 0.67 µM; forward2: TGGGGCTCGACTAGAGGAT, 0.67 µM; reverse: CCCATCCAGCTACAGCTGT, 0.67 µM); 25-µl PCRs were set up using 5× Green GoTaq reaction buffer (Promega; catalog #M7123), the aforementioned primers, the extracted DNA, BioReady rTaq DNA Polymerase (Bulldog Bio, catalog #BSA12L050), and nuclease-free water. The reactions were run using a touchdown protocol on a Peltier Thermal Cycler (MJ Research; PTC-100). The reaction conditions started with a 4-min denaturing step at 94°C, followed by a 16-cycle touchdown, where each cycle starts with a 1-min denaturing step at 94°C, followed by a 1-min annealing step starting at 67°C and decreasing by 1°C each cycle, and a 1-min extension at 72°C. Following the 16 touchdown cycles were 16 additional cycles structured in the same way, except that the annealing temperature was constant at 52°C. Thermal cycling terminated with a 10-min extension step at 72°C, followed by holding at 4°C. PCR products were then run on a 2% agarose gel in TBE buffer. Gel images were processed in ImageJ to measure relative band intensity of the two PCR products. Recombined band intensities were quantified from neural tissue collected from the restored mice as follows: (recombined (287 bp) product – gel background)/(non-recombined (180 bp) product – background). Quantified band intensities were compared between the medial-basal hypothalamus and the dorsal striatum of restored mice, using a paired *t* test.

### RNA extraction, cDNA synthesis, and qRT-PCR

RNA was extracted from one of the bilateral 2-mm^3^ blocks of the medial-basal hypothalamus and analyzed for *Pomc* transcript expression using qRT-PCR. RNA was extracted from hypothalamic samples homogenized by trituration in 50 µl of TRIzol reagent (ThermoFisher Scientific, Ambion, Life Technologies; catalog #15596). Following extraction, the RNA samples were treated with a TURBO DNA-*free* kit (ThermoFisher Scientific, Ambion, Life Technologies; catalog #AM1907) to remove residual genomic DNA. Then, 500 ng of each RNA sample was converted to a 20-µl cDNA library using the GoScript Reverse Transcription System (Promega; catalog #A5000), after which the libraries were diluted 1:4 in nuclease-free water.

A total of 20 µl qPCRs were set up using 2× SYBR Green PCR Master Mix (ThermoFisher Scientific, Applied Biosystems; catalog #4309155), and *Pomc*-transcript or *Ppia*-transcript primers, with 2 µl of diluted cDNA, and nuclease-free water. The *Pomc* primers were designed to span exons 2 and 3 of splice variant 1 (forward: GAGCTGGTGCCTGGAGAG, 300 nM; reverse: TTTTCAGTCAGGGGCTGTTC, 300 nM). The *Ppia* primers were designed to span exons 1 and 3 of all splice variants (forward: CACCGTGTTCTTCGACATCA, 300 nM; reverse: CAGTGCTCAGAGCTCGAAAGT, 300 nM). The reactions were performed in duplicate and loaded onto a MicroAmp Fast Optical 96-well reaction plate (ThermoFisher Scientific, Applied Biosystems; catalog #4346906) and run on a StepOnePlus Real-Time PCR System (ThermoFisher Scientific, Applied Biosystems; catalog #4376600). The reaction conditions started with a 10-min denaturing step at 95°C, followed by 40 cycles of a two-step PCR protocol with a 15-s 95°C denaturing step and a 1-min 60°C annealing step.

C_T_ values were determined by manually setting the threshold at 1, which was in the middle of the exponential phase of amplification for each sample. Baseline readings were automatically assessed by the StepOne Software (ThermoFisher Scientific, Applied Biosystems). Standard curves for each transcript were established by pooling equal amounts of cDNA from all control samples and making serial dilutions (1:1, 1:4, 1:16, 1:64, 1:256, and 1:1024). The percent dilution was Log_10_ transformed (e.g., 1:4 = 25% and Log_10_(25) = 1.398%) and plotted against its respective C_T_ value. The slope and y-intercept of the line formed between all of the dilutions were used to evaluate the relative Log_10_ copy number for each sample (i.e., Log_10_ copy number = [sample C_T_ – Y intercept]/slope), which was then linearized. For each sample, the *Pomc* and *Ppia* linear copy numbers were averaged across duplicates and the *Pomc* average was divided by the *Ppia* average, to yield a normalized *Pomc* expression value. The efficiency of amplification was >90%. *Pomc* expression values for each sample were then standardized to the group average of the control animals to generate a relative quantification of *Pomc* transcript expression. Due to the number of samples, male and female samples were run independently on separate plates with the same standard dilutions, and then normalized within sex.

### IHC

Sucrose-equilibrated brains, preparation described above, were cryosectioned at 30 µm and collected in triplicate with a freezing stage sliding microtome (Leica Biosystems; SM 2010R) into PBS. The sections were then incubated with antisera to POMC [1:1000 (control and restored mice) or 1:10,000 (*Vglut2*-tdTomato animals); Phoenix; rabbit; H-029-30]. Following triplicate washes, one set of the sections from control and restored mice was incubated with a biotinylated goat anti-rabbit secondary antisera (1:500, Vector Labs; catalog #BA-1000 ROS23) followed by treatment with a Vectastain ABC HRP kit (Vector Labs; catalog #PK-4000) and development of a colorimetric stain with diaminobenzidine (250 μg/ml in TBS with 0.1% H_2_O_2_). A second set of sections from control and restored mice was incubated with Alexa Fluor 568 (A568) goat anti-rabbit secondary antibody (1:500; ThermoFisher Scientific; catalog #A-11036). *Vglut2*-tdTomato brain sections were incubated with Alexa Fluor 488 (A488) goat anti-rabbit secondary antibody (1:500; ThermoFisher Scientific; catalog #A-11034).

### IHC image analysis

Images from the DAB-treated IHC tissue sections were acquired with a 6.3×/0.20 160 NPL Fluotar objective on a Leitz Dialux 22 microscope with a Leica DFC280 camera (Leitz/Leica). Fluorescent images used for the *Pomc*-restoration and tdTomato-POMC overlap counts were taken with a 10× objective on a Nikon Eclipse 90i digital upright microscope with a Photometrics CoolSNAP HQ2 CCD Camera. The images were acquired using a 1-s exposure for the POMC signal and a 50-ms exposure time for the tdTomato signal. Resulting tdTomato pictures were then double-processed with ImageJ (http://imagej.nih.gov/ij/), by adjusting the brightness and contrast, to account for different background fluorescence levels in the GABA-rich basomedial ARC and in the glutamate-rich lateral ARC. The representative image shown was acquired using a Nikon A1 confocal microscope.

### Hybridization probes

Riboprobes for ISH were generated from cDNA sequences as follows (NCBI GenBank accession numbers in parenthesis): mouse *Pomc*, bases 502–1008 (short probe) or bases 1–1008 (long probe; NM_001278584.1); *Vglut2*, bases 1762–2390 (NM_080853.3); and *Gad67*, bases 317–892 (NM_008077.4). The *Vglut2* and *Gad67* plasmid templates are a gift from Dr. Erik Hrabovszky (Institute of Experimental Medicine, Budapest), the long *Pomc* template was synthesized by Genscript. For dual-label ISH, the short *Pomc* probe was labeled with digoxigenin-11-UTP (Roche Applied Sciences), and the *Vglut2* and *Gad67* probes with [^35^S]-uridine 5’-(α-thio) triphosphate (PerkinElmer). For triple-label ISH, the long *Pomc* probe was labeled with fluorescein-12-UTP (Roche), the *Gad67* probe with digoxigenin-11-UTP, and the *Vglut2* probe [^35^S]-uridine 5’-(α-thio) triphosphate.

### Dual-label ISH

Sixteen-micrometer coronal sections were cut through the rostrocaudal extent of the hypothalamic ARC using a Leica CM3050 S cryostat, thaw-mounted on Fisherbrand Superfrost Plus Microscope Slides (ThermoFisher Scientific; catalog #12-550-15), air-dried and stored at –80°C. The mounted sections were fixed with 4% PFA in 0.1 M phosphate buffer (pH 7.4) for 20 min, rinsed in PBS for 5 min, acetylated with 0.25% acetic anhydride in 0.1 M triethanolamine for 10 min, treated with ascending ethanol series and chloroform (10 min), partially rehydrated in 95% ethanol, and then processed for hybridization. Two adjacent series of sections, each containing every 7th section, were hybridized with the mixture of the digoxigenin-labeled short *Pomc* riboprobe and the [^35^S]-labeled riboprobe for either *Vglut2* or *Gad67* (diluted to 50,000 cpm/μl) overnight at 56°C in a humidified chamber. The *Pomc* probe was detected with peroxidase-conjugated anti-digoxigenin antibody (diluted 1:100 in 1% blocking reagent, Roche), amplified using the TSA Biotin Tyramide system (PerkinElmer) for 30 min and labeled with Alexa Fluor 488-conjugated streptavidin (diluted 1:500 in 1% blocking reagent, Life Technologies) for 2 h. To detect the radiolabeled *Vglut2* or *Gad67* probe, sections were then rinsed in PBS, dehydrated, air-dried, and coated with Kodak NTB autoradiography emulsion (Carestream Health Inc.). The autoradiograms were developed using Kodak D19 developer after 7 d (*Gad67*) or 12 d (*Vglut2*) of exposure.

### Triple-label ISH

Triple-label ISH was performed on serial hypothalamic sections of three male and three female wild-type C57BL/6J mice, euthanized on postnatal day 65 by decapitation under deep ketamine/xylazine anesthesia. Tissue collection and processing was identical to the dual-label ISH procedure. Sections were hybridized with the mix of the fluorescein-labeled long *Pomc*, digoxigenin-labeled *Gad67*, and [^35^S]-labeled *Vglut2* riboprobes. Following hybridization, sections were first incubated in the peroxidase-conjugated sheep anti-digoxigenin antibody, and the signal was amplified with the TSA Plus DIG kit (catalog #NEL748E001KT, PerkinElmer) for 30 min, using the DIG amplification reagent at 1:500 dilution in 0.05 M Tris (pH 7.6) containing 0.01% H_2_O_2_. Sections were then incubated in a rabbit monoclonal anti-digoxigenin antibody (ThermoFisher, catalog #700772; at 1-μg/ml concentration) for 3 h, in the presence of 2% sodium azide to inactivate peroxidase activity. Sections were thoroughly washed in PBS, and incubated overnight in peroxidase-conjugated sheep anti-fluorescein antibody (Roche, catalog #11426346910; diluted 1:100 in 1% blocking reagent). Signal amplification was applied for 30 min, using the TSA Plus Biotin kit (PerkinElmer) with the TSA Plus biotin reagent diluted 1:300 in 0.05 M Tris and 0.01% H_2_O_2_. The biotin deposits and the anti-digoxigenin-antibody were detected with Alexa Fluor 488-conjugated streptavidin and Alexa Fluor 594-conjugated anti-rabbit IgG (Jackson ImmunoResearch; 1:200), respectively. The sections were then dehydrated, dipped in Kodak NTB autoradiography emulsion, and developed after 10 d as described above.

### ISH image analysis

Fluorescent signals and darkfield emulsion autoradiography images of the same field were captured with the 10× objective of a Zeiss Axioplan 2 microscope (Carl Zeiss) equipped with a RT SPOT digital camera (Diagnostic Instruments). Every 14th section (five sections per mouse), covering the rostro-caudal extent of the Arc was used to count the number of *Pomc*, dual-labeled *Pomc-Vglut2* and *Pomc-Gad67*, and triple-labeled *Pomc-Vglut2-Gad67* neurons. To be considered specifically labeled with the radioactive *Vglut2* or *Gad67* probes, *Pomc* neurons had to exhibit at least a 5-fold higher silver grain density than over background regions. This was confirmed by ImageJ for each *Pomc* neuron with lighter silver grain label. For publication images, the green fluorescence of Alexa Fluor 488 (*Pomc*) was pseudocolored to either red or blue as noted in the two relevant figure legends to better visualize colocalization with the silver grain autoradiography signal.

### Data analysis

All statistical testing was performed using GraphPad Prism 7.04 for Windows (GraphPad Software). Datasets with an *n* ≥ 8 were tested for normality using the D’Agostino and Pearson normality assessment, while groups with *n* < 8, were assessed with the Shapiro–Wilk normality test. All sample sizes, mean ± SEM, and measured units are located in the descriptive statistics table (Table 1). The data structures, specific statistical tests used, and numerical results are located in statistical tests table (Table 2). Both Tables 1, 2 are organized and labeled according to the respective figure panels representing the data, or when appropriate, annotated as not shown.

**Table 1.** Descriptive statistics

**Figure**	**Sample size**	**Mean ± SEM**	**Units**
1*I*	*n* = 5 (2 Males, 3 Females)	Level 1: 0.52 ± 0.07	Proportion of
		Level 2: 0.48 ± 0.02	POMC neurons
		Level 3: 0.43 ± 0.02	
		Level 4: 0.45 ± 0.03	
		Level 5: 0.48 ± 0.05	
			
2*C*	*n* = 9	Hypothalamus: 0.51 ± 0.03	Arbitrary intensity units
Restored Group		Striatum: 0.12 ± 0.01	
			
3*A*	Control: *n* = 14	Data order: control, FNΔ2, restored	Grams
Males	FNΔ2: *n* = 8	Week 3: 9.8 ± 0.6, 11.7 ± 0.7, 10.7 ± 0.4	
	Restored: *n* = 22	Week 4: 14.4 ± 0.9, 16.4 ± 0.8, 15.4 ± 0.5	
		Week 5: 19.2 ± 0.8, 21.8 ± 1.0, 19.7 ± 0.4	
		Week 6: 21.7 ± 0.6, 25.2 ± 1.3, 21.7 ± 0.3	
		Week 7: 23.2 ± 0.5, 27.6 ± 1.3, 23.1 ± 0.4	
		Week 8: 24.6 ± 0.5, 30.2 ± 1.7, 24.2 ± 0.4	
		Week 9: 25.4 ± 0.5, 32.7 ± 1.7, 24.9 ± 0.4	
		Week 10: 26.9 ± 0.5, 35.1 ± 1.8, 25.7 ± 0.5	
		Week 11: 27.5 ± 0.5, 37.3 ± 1.8, 26.4 ± 0.5	
		Week 12: 28.3 ± 0.5, 39.7 ± 2.0, 27.0 ± 0.4	
3*B*	Control: *n* = 12	Data order: control, FNΔ2, restored	Grams
Females	FNΔ2: *n* = 6	Week 3: 9.8 ± 0.4, 10.9 ± 0.9, 9.6 ± 0.3	
	Restored: *n* = 17	Week 4: 13.4 ± 0.6, 14.8 ± 1.2, 12.8 ± 0.4	
		Week 5: 16.6 ± 0.5, 19.5 ± 1.2, 15.6 ± 0.3	
		Week 6: 17.5 ± 0.5, 23.0 ± 1.1, 16.6 ± 0.3	
		Week 7: 18.2 ± 0.6, 24.2 ± 1.1, 17.3 ± 0.2	
		Week 8: 19.2 ± 0.7, 26.9 ± 1.3, 17.9 ± 0.3	
		Week 9: 18.6 ± 0.4, 29.0 ± 1.6, 18.5 ± 0.3	
		Week 10: 19.6 ± 0.5, 31.1 ± 1.9, 19.0 ± 0.3	
		Week 11: 20.1 ± 0.5, 34.3 ± 2.2, 19.5 ± 0.3	
		Week 12: 20.4 ± 0.5, 36.1 ± 2.7, 20.0 ± 0.3	
3*C*	Control: *n* = 8	1.55 ± 0.11	Grams
Fat mass, males	FNΔ2: *n* = 8	14.08 ± 1.12	
	Restored: *n* = 10	1.52 ± 0.11	
3*C*	Control: *n* = 8	21.36 ± 0.27	Grams
Lean mass, males	FNΔ2: *n* = 8	21.66 ± 0.90	
	Restored: *n* = 10	21.20 ± 0.44	
3*D*	Control: *n* = 7	1.46 ± 0.13	Grams
Fat mass, females	FNΔ2: *n* = 4	13.08 ± 1.46	
	Restored: *n* = 10	1.60 ± 0.15	
3*D*	Control: *n* = 7	15.67 ± 0.28	Grams
Lean mass, females	FNΔ2: *n* = 4	17.45 ± 1.21	
	Restored: *n* = 10	14.88 ± 0.28	
3*E*	Control: *n* = 9	339 ± 11	Milligrams
Gonadal fat, males	FNΔ2: *n* = 4	2111 ± 230	
	Restored: *n* = 10	308 ± 18	
3*E*	Control: *n* = 9	189 ± 17	Milligrams
Inguinal fat, males	FNΔ2: *n* = 4	1441 ± 97	
	Restored: *n* = 9	206 ± 7	
3*F*	Control: *n* = 7	221 ± 28	Milligrams
Gonadal fat, females	FNΔ2: *n* = 3	2191 ± 243	
	Restored: *n* = 11	257 ± 33	
3*F*	Control: *n* = 7	206 ± 14	Milligrams
Inguinal fat, females	FNΔ2: *n* = 3	1532 ± 327	
	Restored: *n* = 10	192 ± 15	
4*F*	Three sections/animal		POMC neurons/section
	Control, animals		
	A568: *n* = 3 (1 Male, 2 Females)	82.11 ± 6.67	
	DAB: *n* = 4 (2 Males, 2 Females)	122.40 ± 7.04	
	Restored, animals		
	A568: *n* = 3 (2 Males, 1 Female)	69.22 ± 6.64	
	DAB: *n* = 3 (2 Males, 1 Female)	129.30 ± 8.87	
4*F*	Three sections/animal A568: *n* = 6 (3 Males, 3 Females) DAB: *n* = 7 (4 Males, 3 Females) A488: *n* = 5 (2 Males, 3 Females)	75.67 ± 4.83 125.4 ± 5.45 100.5 ± 4.88	POMC neurons/section

5 (data not shown)	Control: *n* = 6 (4 Males, 2 Females)	606.30 ± 7.28	Number of *Pomc* neurons
number of Pomc neurons	Restored: *n* = 9 (6 Males, 3 Females)	407.10 ± 14.19	
5*E*	Control: *n* = 6 (4 Males, 2 Females)	*Vglut2* ^+^: 55.77 ± 1.86	% of *Pomc* neurons
		*Gad67* ^+^: 64.63 ± 0.90	
	Restored: *n* = 8 (5 Males, 3 Females)	*Vglut2* ^+^: 65.80 ± 1.95	
		*Gad67* ^+^: 60.59 ± 1.29	
5*E* (data not shown)	Control: *n* = 6 (4 Males, 2 Females)	–8.87 ± 2.21	%*Vglut2* ^+^, %*Gad67* ^+^
Difference	Restored: *n* = 8 (5 Males, 3 Females)	5.21 ± 2.14	
5*F*	Control: *n* = 6 (4 Males, 2 Females)	*Vglut2* ^+^: 338.30 ± 13.65	Number of *Pomc* neurons
		*Gad67* ^+^: 391.80 ± 4.21	
	Restored: *n* = 8 (5 Males, 3 Females)	*Vglut2* ^+^: 270.50 ± 12.44	
		*Gad67* ^+^: 250.10 ± 12.95	
5*F* (data not shown)	Control: *n* = 6 (4 Males, 2 Females)	–53.5 ± 12.92	#*Vglut2* ^+^, #*Gad67* ^+^
Difference	Restored: *n* = 8 (5 Males, 3 Females)	20.38 ± 8.87	
5*G*	Control: *n* = 16 (10 Males, 6 Females)	1.00 ± 0.10	Relative *Pomc* expression
	FNΔ2: *n* = 4 (1 Male, 3 Females)	0.03 ± 0.00	
	Restored: *n* = 15 (7 Males, 8 Females)	1.07 ± 0.10	
			
6*M*	*n* = 6 (3 Males, 3 Females)	Level 1: 16.73 ± 3.10	% of *Pomc* neurons
		Level 2: 25.04 ± 1.41	
		Level 3: 19.90 ± 1.33	
		Level 4: 22.78 ± 2.39	
		Level 5: 15.55 ± 1.04	
6*N*	*n* = 6 (3 Males, 3 Females)	*Pomc*-only: 7.05 ± 1.10	% of *Pomc* neurons
		*Gad67* ^+^: 34.71 ± 2.42	
		*Vglut2* ^+^: 20.62 ± 1.31	
		*Vglut2/Gad67* ^+^: 37.61 ± 1.97	
6*O*	*n* = 6 (3 Males, 3 Females)	*Pomc*-only:	% of *Pomc* neurons
		Level 1: 2.37 ± 0.47	
		Level 2: 1.62 ± 0.37	
		Level 3: 1.28 ± 0.18	
		Level 4: 1.30 ± 0.62	
		Level 5: 0.48 ± 0.35	
		*Gad67* ^+^:	
		Level 1: 5.44 ± 1.80	
		Level 2: 10.26 ± 0.52	
		Level 3: 8.80 ± 0.71	
		Level 4: 6.47 ± 0.76	
		Level 5: 3.75 ± 0.70	
		*Vglut2* ^+^:	
		Level 1: 6.22 ± 0.86	
		Level 2: 6.07 ± 1.20	
		Level 3: 3.12 ± 0.31	
		Level 4: 3.90 ± 0.22	
		Level 5: 1.31 ± 0.29	
		*Vglut2/Gad67* ^+^:	
		Level 1: 2.69 ± 0.54	
		Level 2: 7.09 ± 1.00	
		Level 3: 6.71 ± 0.91	
		Level 4: 11.12 ± 1.58	
		Level 5: 10.02 ± 0.44	
6*P* Linear regression analysis	*n* = 6 (3 Males, 3 Females)	*Pomc*-only: y = –6.795x + 40.39	y = mx + b
		*Gad67* ^+^: y = –1.977x + 25.93	
		*Gad67* ^+^ (2–5): y = –6.449x + 43.82	
		*Vglut2* ^+^: y = –5.784x + 37.35	
		*Vglut2/Gad67* ^+^: y = 4.955x + 5.14	

**Table 2.** Statistical tests table

**Figure**	**Data structure**	**Type of test**	**Statistical data**
1*I*	Normal	One-way RM ANOVA	Level: *F*_(1.65,6.60)_ = 1.225, *p* = 0.34
	distribution	(Geisser-Greenhouse correction)	Animal: *F*_(4,16)_ = 5.038, *p* = 8.04e-3
			Tukey’s multiple comparisons test
			Level 1 vs level 2, *q* = 1.04, *p* = 0.94
			Level 1 vs level 3, *q* = 2.11, *p* = 0.62
			Level 1 vs level 4, *q* = 1.80, *p* = 0.72
			Level 1 vs level 5, *q* = 1.07, *p* = 0.93
			Level 2 vs level 3, *q* = 10.01, *p* = 9.83e-3
			Level 2 vs level 4, *q* = 5.23, *p* = 8.90e-2
			Level 2 vs level 5, *q* = 0.25, *p* = 0.99
			Level 3 vs level 4, *q* = 2.68, *p* = 0.44
			Level 3 vs level 5, *q* = 1.57, *p* = 0.80
			Level 4 vs level 5, *q* = 1.21, *p* = 0.90
Figure	Data structure	Type of test	Statistical data
2*C*	Normal	Paired *t* test	*t* = 11.98, df = 8, *p* = 2.17e-6
Restored group	distribution		
Figure	Data structure	Type of test	Statistical data
3*A*	Growth curve	Two-way ANOVA	Interaction *F*_(18,384)_ = 8.76, *p* < 1.00e-15
Males			Time: *F*_(9,384)_ = 244, *p* < 1.00e-15
			Genotype: *F*_(2,384)_ = 198.2, *p* < 1.00e-15
			Tukey’s multiple comparisons test
			Five weeks:
			Control vs FNΔ2, *q* = 3.23, *p* = 5.97e-2
			Control vs restored, *q* = 0.80, *p* = 0.84
			FNΔ2 vs restored, *q* = 2.80, *p* = 0.12
			Six weeks:
			Control vs FNΔ2, *q* = 4.34, *p* = 6.46e-3
			Control vs restored, *q* = 0.00, *p* > 0.99
			FNΔ2 vs restored, *q* = 4.66, *p* = 3.07e-3
			Seven weeks:
			Control vs FNΔ2, *q* = 5.46, *p* = 3.90e-4
			Control vs restored, *q* = 0.16, *p* = 0.99
			FNΔ2 vs restored, *q* = 5.99, *p* = 8.38e-5
3*B*	Growth curve	Two-way ANOVA	Interaction *F*_(18,294)_ = 19.75, *p* < 1.00e-15
Females			Time: *F*_(9,294)_ = 158.4, *p* < 1.00e-15
			Genotype: *F*_(2,294)_ = 473.2, *p* < 1.00e-15
			Tukey’s multiple comparisons test
			Four weeks:
			Control vs FNΔ2, *q* = 1.98, *p* = 0.34
			Control vs restored, *q* = 1.13, *p* = 0.71
			FNΔ2 vs restored, *q* = 2.99, *p* = 8.94e-2
			Five weeks:
			Control vs FNΔ2, *q* = 4.11, *p* = 1.10e-2
			Control vs restored, *q* = 1.88, *p* = 0.38
			FNΔ2 vs restored, *q* = 5.82, *p* = 1.48e-4
			Six weeks:
			Control vs FNΔ2, *q* = 7.80, *p* = 2.32e-7
			Control vs restored, *q* = 1.69, *p* = 0.46
			FNΔ2 vs restored, *q* = 9.55, *p* = 2.31e-10
3*C*	Normal	One-way ANOVA	*F*_(2,23)_ = 138.4, *p* < 15.1e-14
Fat mass, males	distribution		Tukey’s multiple comparisons test
			Control vs FNΔ2, *q* = 19.97, *p* = 2.39e-12
			Control vs restored, *q* = 0.05, *p* > 0.99
			FNΔ2 vs restored, *q* = 21.10, *p* = 7.78e-13
3*C*	Normal	One-way ANOVA	*F*_(2,23)_ = 0.167, *p* = 0.85
Lean mass, males	distribution		Tukey’s multiple comparisons test
			Control vs FNΔ2, *q* = 0.50, *p* = 0.93
			Control vs restored, *q* = 0.29, *p* = 0.98
			FNΔ2 vs restored, *q* = 0.81, *p* = 0.84
3*D*	Normal	One-way ANOVA	*F*_(2,18)_ = 138.4, *p* = 1.18e-11
Fat mass, females	distribution		Tukey’s multiple comparisons test
			Control vs FNΔ2, *q* = 21.01, *p* = 4.51e-11
			Control vs restored, *q* = 0.33, *p* = 0.97
			FNΔ2 vs restored, *q* = 21.98, *p* = 2.09e-12

3*D*	Normal	One-way ANOVA	*F*_(2,18)_ = 6.16, *p* = 9.17e-3
Lean mass, females	distribution		Tukey’s multiple comparisons test
			Control vs FNΔ2, *q* = 3.24, *p* = 0.08
			Control vs restored, *q* = 1.83, *p* = 0.42
			FNΔ2 vs restored, *q* = 4.96, *p* = 6.74e-3
3*E*	Normal	One-way ANOVA	*F*_(2,20)_ = 157.4, *p* = 5.80e-14
Gonadal fat, males	distribution		Tukey’s multiple comparisons test
			Control vs FNΔ2, *q* = 22.76, *p* = 2.00e-12
			Control vs restored, *q* = 0.51, *p* = 0.93
			FNΔ2 vs restored, *q* = 23.51, *p* = 1.09e-12
3*E*	Normal	One-way ANOVA	*F*_(2,19)_ = 354.5, *p* < 1.00e-15
Inguinal fat, males	distribution		Tukey’s multiple comparisons test
			Control vs FNΔ2, *q* = 34.87, *p* < 1.00e-15
			Control vs restored, *q* = 0.60, *p* = 0.91
			FNΔ2 vs restored, *q* = 34.40, *p* < 1.00e-15
3*F*	Normal	One-way ANOVA	*F*_(2,18)_ = 173.8, *p* = 1.70e-12
Gonadal fat, females	distribution		Tukey’s multiple comparisons test
			Control vs FNΔ2, *q* = 24.08, *p* = 4.26e-12
			Control vs restored, *q* = 0.62, *p* = 0.90
			FNΔ2 vs restored, *q* = 25.06, *p* = 2.12e-12
3*F*	Normal	One-way ANOVA	*F*_(2,17)_ = 57.55, *p* = 2.70e-8
Inguinal fat, females	distribution		Tukey’s multiple comparisons test
			Control vs FNΔ2, *q* = 13.68, *p* = 7.26e-8
			Control vs restored, *q* = 0.20, *p* = 0.99
			FNΔ2 vs restored, *q* = 14.49, *p* = 3.12e-8
4*F*	Normal	Two-way ANOVA	Interaction *F*_(1,36)_ = 3.207, *p* = 0.11
	distribution		Genotype: *F*_(1,36)_ = 3.61e-5, *p* = 0.99
			Detection: *F*_(1,36)_ = 45.97, *p* = 6.39e-8
			Tukey’s multiple comparisons test
			Control-A568 vs restored-A568, *q* = 1.57, *p* = 0.69
			Control-A568 vs control-DAB, *q* = 5.24, *p* = 3.76e-3
			Control-A568 vs restored-DAB, *q* = 6.65, *p* = 2.10e-4
			Restored-A568 vs control-DAB, *q* = 6.92, *p* = 1.19e-4
			Restored-A568 vs restored-DAB, *q* = 8.26, *p* = 6.60e-6
			Control-DAB vs restored-DAB, *q* = 1.74, *p* = 0.61
4*F*	Normal distribution	One-way ANOVA	*F*_(2,51)_ = 24.9, *p* = 2.85e-8Tukey’s multiple comparisons testA568 vs DAB, *q* = 9.97, *p* = 1.35e-8A548 vs A488, *q* = 4.58, *p* = 5.87e-3DAB vs A488, *q* = 4.74, *p* = 4.31e-3
			
5 (data not shown)	Normal	Student’s *t* test	*t* = 10.75, df = 13, *p* = 7.76e-8
# of *Pomc* neurons	distribution		
5*E*	Normal	Two-way RM ANOVA	Interaction *F*_(1,12)_ = 20.23, *p* = 7.30e-4
	distribution		Genotype: *F*_(1,12)_ = 3.181, *p* = 9.98e-2
			Marker: *F*_(1,12)_ = 1.363, *p* = 0.27
			Sidak’s multiple comparisons test
			Control-*Vglut2* ^+^ vs restored-*Vglut2* ^+^, *t* = 4.37, *p* = 4.10e-4
			Control-*Gad67* ^+^ vs restored-*Gad67* ^+^, *t* = 1.76, *p* = 0.17
			Control-*Vglut2* ^+^ vs control-*Gad67* ^+^, *t* = 3.75, *p* = 5.56e-3
			Restored-*Vglut2* ^+^ vs restored-*Gad67* ^+^, *t* = 2.54, *p* = 5.09e-2
5*E* (data not shown)	Normal	Student’s *t* test	*t* = 4.498, df = 12, *p* = 7.30e-4
Difference	distribution		
5*F*	Normal	Two-way RM ANOVA	Interaction *F*_(1,12)_ = 23.86, *p* = 3.76e-4
	distribution		Genotype: *F*_(1,12)_ = 46.4, *p* = 1.87e-5
			Marker: *F*_(1,12)_ = 4.797, *p* = 4.90e-2
			Sidak’s multiple comparisons test
			Control-*Vglut2* ^+^ vs restored-*Vglut2* ^+^, *t* = 3.96, *p* = 1.17e-3
			Control-*Gad67* ^+^ vs restored-*Gad67* ^+^, *t* = 8.27, *p* = 3.50e-8
			Control-*Vglut2* ^+^ vs control-*Gad67* ^+^, *t* = 4.68, *p* = 1.07e-3
			Restored-*Vglut2* ^+^ vs restored-*Gad67* ^+^, *t* = 2.06, *p* = 0.12
5*E* (data not shown)	Normal	Student’s *t* test	*t* = 4.884, df = 12, *p* = 3.76e-4
Difference	distribution		

5*G*	Normal	One-way ANOVA	*F*_(2,32)_ = 10.63, *p* = 2.88e-4
	distribution		Tukey’s multiple comparisons test
			Control vs FNΔ2, *q* = 5.96, *p* = 5.43e-4
			Control vs restored, *q* = 0.65, *p* = 0.89
			FNΔ2 vs restored, *q* = 6.34, *p* = 2.54e-4
6*M*	Normal	Two-way RM ANOVA	Interaction *F*_(12,60)_ = 13.10, *p* = 1.19e-12
	distribution		Neurotransmitter: *F*_(3,15)_ = 47.14, *p* = 7.16e-8
			Level: *F*_(4,20)_ = 3.16, *p* = 3.63e-2
			Tukey’s multiple comparisons test
			Level 1 vs level 2, *q* = 3.71, *p* = 0.10
			Level 1 vs level 3, *q* = 1.42, *p* = 0.85
			Level 1 vs level 4, *q* = 2.70, *p* = 0.35
			Level 1 vs level 5, *q* = 0.52, *p* > 0.99
			Level 2 vs level 3, *q* = 2.29, *p* = 0.50
			Level 2 vs level 4, *q* = 1.01, *p* = 0.95
			Level 2 vs level 5, *q* = 4.23, *p* = 5.02e-2
			Level 3 vs level 4, *q* = 1.28, *p* = 0.89
			Level 3 vs level 5, *q* = 1.94, *p* = 0.65
			Level 4 vs level 5, *q* = 3.22, *p* = 0.19
6*N*	Normal	Two-way RM ANOVA	Interaction *F*_(12,60)_ = 13.10, *p* = 1.19e-12
	distribution		Neurotransmitter: *F*_(3,15)_ = 47.14, *p* = 7.16e-8
			Level: *F*_(4,20)_ = 3.16, *p* = 3.63e-2
			Tukey’s multiple comparisons test
			*Vglut2* ^+^ vs *Gad67* ^+^, *q* = 6.87, *p* = 1.07e-3
			*Vglut2* ^+^ vs *Vglut2/Gad67* ^+^, *q* = 8.29, *p* = 1.66e-4
			*Vglut2* ^+^ vs *Pomc*-only, *q* = 6.62, *p* = 1.51e-3
			*Gad67* ^+^ vs *Vglut2/Gad67* ^+^, *q* = 1.42, *p* = 0.75
			*Gad67* ^+^ vs *Pomc*-only, *q* = 13.49, *p* = 5.09e-7
			*Vglut2/Gad67* ^+^ vs *Pomc*-only, *q* = 14.90, *p* = 1.37e-7
6*O*	Normal	Two-way RM ANOVA	Interaction *F*_(12,60)_ = 13.10, *p* = 1.19e-12
	distribution		Neurotransmitter: *F*_(3,15)_ = 47.14, *p* = 7.16e-8
			Level: *F*_(4,20)_ = 3.16, *p* = 3.63e-2
			Tukey’s multiple comparisons test
			*Vglut2* ^+^:
			Level 1 vs level 2, *q* = 0.23, *p* = 0.99
			Level 1 vs level 3, *q* = 4.54, *p* = 1.75e-2
			Level 1 vs level 4, *q* = 3.39, *p* = 0.13
			Level 1 vs level 5, *q* = 7.18, *p* = 3.80e-5
			Level 2 vs level 3, *q* = 4.31, *p* = 2.71e-2
			Level 2 vs level 4, *q* = 3.16, *p* = 0.18
			Level 2 vs level 5, *q* = 6.96, *p* = 6.80e-5
			Level 3 vs level 4, *q* = 1.15, *p* = 0.93
			Level 3 vs level 5, *q* = 2.64, *p* = 0.35
			Level 4 vs level 5, *q* = 3.79, *p* = 6.85e-2
			*Gad67* ^+^:
			Level 1 vs level 2, *q* = 7.05, *p* = 5.33e-5
			Level 1 vs level 3, *q* = 4.90, *p* = 8.36e-3
			Level 1 vs level 4, *q* = 1.50, *p* = 0.83
			Level 1 vs level 5, *q* = 7.18, *p* = 0.41
			Level 2 vs level 3, *q* = 2.15, *p* = 0.55
			Level 2 vs level 4, *q* = 5.55, *p* = 2.03e-3
			Level 2 vs level 5, *q* = 9.53, *p* = 6.85e-8
			Level 3 vs level 4, *q* = 3.40, *p* = 0.13
			Level 3 vs level 5, *q* = 7.38, *p* = 2.26e-5
			Level 4 vs level 5, *q* = 3.98, *p* = 4.98e-2
			*Pomc*-only:
			Level 1 vs level 2, *q* = 1.10, *p* = 0.94
			Level 1 vs level 3, *q* = 1.59, *p* = 0.79
			Level 1 vs level 4, *q* = 1.56, *p* = 0.80
			Level 1 vs level 5, *q* = 2.77, *p* = 0.30
			Level 2 vs level 3, *q* = 0.49, *p* = 0.99
			Level 2 vs level 4, *q* = 0.46, *p* = 0.99
			Level 2 vs level 5, *q* = 1.67, *p* = 0.76
			Level 3 vs level 4, *q* = 0.03, *p* = 0.99
			Level 3 vs level 5, *q* = 1.18, *p* = 0.92
			Level 4 vs level 5, *q* = 1.21, *p* = 0.91

			*Vglut2/Gad67* ^+^:
			Level 1 vs level 2, *q* = 6.44, *p* = 2.51e-4
			Level 1 vs level 3, *q* = 5.88, *p* = 9.63e-4
			Level 1 vs level 4, *q* = 12.31, *p* = 5.13e-11
			Level 1 vs level 5, *q* = 10.71, *p* = 2.63e-9
			Level 2 vs level 3, *q* = 0.56, *p* = 0.99
			Level 2 vs level 4, *q* = 5.87, *p* = 9.77e-4
			Level 2 vs level 5, *q* = 4.27, *p* = 2.92e-2
			Level 3 vs level 4, *q* = 6.43, *p* = 5.54e-4
			Level 3 vs level 5, *q* = 4.83, *p* = 9.68e-3
			Level 4 vs level 5, *q* = 1.60, *p* = 0.79
			Level 1:
			*Vglut2* ^+^ vs *Gad67* ^+^, *q* = 1.14, *p* = 0.85
			*Vglut2* ^+^ vs *Vglut2/Gad67* ^+^, *q* = 5.16, *p* = 3.00e-3
			*Vglut2* ^+^ vs *Pomc*-only, *q* = 5.63, *p* = 1.05e-3
			*Gad67* ^+^ vs *Vglut2/Gad67* ^+^, *q* = 4.02, *p* = 3.02e-2
			*Gad67* ^+^ vs *Pomc*-only, *q* = 4.49, *p* = 1.23e-2
			*Vglut2/Gad67* ^+^ vs *Pomc*-only, *q* = 0.47, *p* = 0.99
			Level 2:
			*Vglut2* ^+^ vs *Gad67* ^+^, *q* = 6.14, *p* = 3.18e-4
			*Vglut2* ^+^ vs *Vglut2/Gad67* ^+^, *q* = 1.50, *p* = 0.72
			*Vglut2* ^+^ vs *Pomc*-only, *q* = 6.51, *p* = 1.29e-4
			*Gad67* ^+^ vs *Vglut2/Gad67* ^+^, *q* = 4.64, *p* = 9.16e-3
			*Gad67* ^+^ vs *Pomc*-only, *q* = 12.64, *p* = 2.74e-11
			*Vglut2/Gad67* ^+^ vs *Pomc*-only, *q* = 8.00, *p* = 2.67e-6
			Level 3:
			*Vglut2* ^+^ vs *Gad67* ^+^, *q* = 8.30, *p* = 1.20e-6
			*Vglut2* ^+^ vs *Vglut2/Gad67* ^+^, *q* = 5.25, *p* = 2.48e-3
			*Vglut2* ^+^ vs *Pomc*-only, *q* = 2.68, *p* = 0.24
			*Gad67* ^+^ vs *Vglut2/Gad67* ^+^, *q* = 3.05, *p* = 0.15
			*Gad67* ^+^ vs *Pomc*-only, *q* = 10.99, *p* = 7.50e-10
			*Vglut2/Gad67* ^+^ vs *Pomc*-only, *q* = 7.94, *p* = 3.19e-6
			Level 4:
			*Vglut2* ^+^ vs *Gad67* ^+^, *q* = 3.75, *p* = 4.92e-2
			*Vglut2* ^+^ vs *Vglut2/Gad67* ^+^, *q* = 10.53, *p* = 2.60e-9
			*Vglut2* ^+^ vs *Pomc*-only, *q* = 3.80, *p* = 4.45e-2
			*Gad67* ^+^ vs *Vglut2/Gad67* ^+^, *q* = 6.78, *p* = 6.41e-5
			*Gad67* ^+^ vs *Pomc*-only, *q* = 7.55, *p* = 8.88e-6
			*Vglut2/Gad67* ^+^ vs *Pomc*-only, *q* = 14.33, *p* = 2.00e-11
			Level 5:
			*Vglut2* ^+^ vs *Gad67* ^+^, *q* = 3.56, *p* = 6.73e-2
			*Vglut2* ^+^ vs *Vglut2/Gad67* ^+^, *q* = 12.73, *p* = 2.58e-11
			*Vglut2* ^+^ vs *Pomc*-only, *q* = 1.22, *p* = 0.83
			*Gad67* ^+^ vs *Vglut2/Gad67* ^+^, *q* = 9.17, *p* = 1.13e-7
			*Gad67* ^+^ vs *Pomc*-only, *q* = 4.78, *p* = 6.89e-3
			*Vglut2/Gad67* ^+^ vs *Pomc*-only, *q* = 13.94, *p* = 2.01e-11
6*P*	Normal	Two-way RM ANOVA	Interaction *F*_(12,60)_ = 11.47, *p* = 1.69e-11
	distribution		Neurotransmitter: *F*_(3,15)_ = 2.105, *p* = 0.14
			Level: *F*_(4,20)_ = 3.205, *p* = 3.46e-2
			Tukey’s multiple comparisons test
			*Vglut2* ^+^:
			Level 1 vs level 2, *q* = 0.45, *p* = 0.99
			Level 1 vs level 3, *q* = 5.48, *p* = 2.42e-3
			Level 1 vs level 4, *q* = 3.95, *p* = 5.22e-2
			Level 1 vs level 5, *q* = 8.61, *p* = 8.65e-7
			Level 2 vs level 3, *q* = 5.02, *p* = 6.49e-3
			Level 2 vs level 4, *q* = 3.50, *p* = 0.11
			Level 2 vs level 5, *q* = 8.16, *p* = 2.92e-6
			Level 3 vs level 4, *q* = 1.52, *p* = 0.82
			Level 3 vs level 5, *q* = 3.13, *p* = 0.19
			Level 4 vs level 5, *q* = 4.65, *p* = 1.39e-2
			*Gad67* ^+^:
			Level 1 vs level 2, *q* = 5.36, *p* = 3.13e-3
			Level 1 vs level 3, *q* = 3.76, *p* = 7.27e-2
			Level 1 vs level 4, *q* = 1.37, *p* = 0.87
			Level 1 vs level 5, *q* = 1.54, *p* = 0.81
			Level 2 vs level 3, *q* = 1.60, *p* = 0.79
			Level 2 vs level 4, *q* = 4.00, *p* = 4.85e-2
			Level 2 vs level 5, *q* = 6.90, *p* = 7.79e-5

			Level 3 vs level 4, *q* = 2.39, *p* = 0.45
			Level 3 vs level 5, *q* = 5.30, *p* = 3.56e-3
			Level 4 vs level 5, *q* = 2.91, *p* = 0.25
			*Pomc*-only:
			Level 1 vs level 2, *q* = 4.81, *p* = 1.02e-2
			Level 1 vs level 3, *q* = 6.05, *p* = 6.39e-4
			Level 1 vs level 4, *q* = 6.88, *p* = 8.36e-5
			Level 1 vs level 5, *q* = 11.14, *p* = 8.14e-9
			Level 2 vs level 3, *q* = 1.24, *p* = 0.90
			Level 2 vs level 4, *q* = 2.07, *p* = 0.59
			Level 2 vs level 5, *q* = 6.33, *p* = 3.27e-4
			Level 3 vs level 4, *q* = 0.83, *p* = 0.98
			Level 3 vs level 5, *q* = 5.09, *p* = 5.66e-3
			Level 4 vs level 5, *q* = 4.26, *p* = 2.98e-2
			*Vglut2/Gad67* ^+^:
			Level 1 vs level 2, *q* = 4.02, *p* = 4.68e-2
			Level 1 vs level 3, *q* = 3.69, *p* = 8.14e-2
			Level 1 vs level 4, *q* = 7.75, *p* = 8.64e-6
			Level 1 vs level 5, *q* = 7.01, *p* = 6.00e-5
			Level 2 vs level 3, *q* = 0.32, *p* = 0.99
			Level 2 vs level 4, *q* = 3.74, *p* = 7.55e-2
			Level 2 vs level 5, *q* = 2.99, *p* = 0.23
			Level 3 vs level 4, *q* = 4.06, *p* = 4.31e-2
			Level 3 vs level 5, *q* = 3.32, *p* = 0.15
			Level 4 vs level 5, *q* = 0.75, *p* = 0.98
			Level 1:
			*Vglut2* ^+^ vs *Gad67* ^+^, *q* = 5.49, *p* = 1.47e-3
			*Vglut2* ^+^ vs *Vglut2/Gad67* ^+^, *q* = 8.19, *p* = 1.62e-6
			*Vglut2* ^+^ vs *Pomc*-only, *q* = 2.08, *p* = 0.46
			*Gad67* ^+^ vs *Vglut2/Gad67* ^+^, *q* = 2.70, *p* = 0.23
			*Gad67* ^+^ vs *Pomc*-only, *q* = 7.56, *p* = 8.58e-6
			*Vglut2/Gad67* ^+^ vs *Pomc*-only, *q* = 10.27, *p* = 5.40e-8
			Level 2:
			*Vglut2* ^+^ vs *Gad67* ^+^, *q* = 0.33, *p* = 0.99
			*Vglut2* ^+^ vs *Vglut2/Gad67* ^+^, *q* = 3.72, *p* = 5.11e-2
			*Vglut2* ^+^ vs *Pomc*-only, *q* = 2.28, *p* = 0.38
			*Gad67* ^+^ vs *Vglut2/Gad67* ^+^, *q* = 4.05, *p* = 2.87e-2
			*Gad67* ^+^ vs *Pomc*-only, *q* = 2.61, *p* = 0.26
			*Vglut2/Gad67* ^+^ vs *Pomc*-only, *q* = 1.44, *p* = 0.74
			Level 3:
			*Vglut2* ^+^ vs *Gad67* ^+^, *q* = 3.75, *p* = 4.90e-2
			*Vglut2* ^+^ vs *Vglut2/Gad67* ^+^, *q* = 0.98, *p* = 0.90
			*Vglut2* ^+^ vs *Pomc*-only, *q* = 1.50, *p* = 0.71
			*Gad67* ^+^ vs *Vglut2/Gad67* ^+^, *q* = 2.77, *p* = 0.21
			*Gad67* ^+^ vs *Pomc*-only, *q* = 2.25, *p* = 0.39
			*Vglut2/Gad67* ^+^ vs *Pomc*-only, *q* = 0.53, *p* = 0.98
			Level 4:
			*Vglut2* ^+^ vs *Gad67* ^+^, *q* = 0.17, *p* = 0.99
			*Vglut2* ^+^ vs *Vglut2/Gad67* ^+^, *q* = 3.51, *p* = 7.26e-2
			*Vglut2* ^+^ vs *Pomc*-only, *q* = 0.85, *p* = 0.98
			*Gad67* ^+^ vs *Vglut2/Gad67* ^+^, *q* = 3.68, *p* = 5.50e-2
			*Gad67* ^+^ vs *Pomc*-only, *q* = 0.68, *p* = 0.96
			*Vglut2/Gad67* ^+^ vs *Pomc*-only, *q* = 4.36, *p* = 1.60e-2
			Level 5:
			*Vglut2* ^+^ vs *Gad67* ^+^, *q* = 1.58, *p* = 0.68
			*Vglut2* ^+^ vs *Vglut2/Gad67* ^+^, *q* = 7.42, *p* = 1.24e-5
			*Vglut2* ^+^ vs *Pomc*-only, *q* = 0.45, *p* = 0.99
			*Gad67* ^+^ vs *Vglut2/Gad67* ^+^, *q* = 5.85, *p* = 6.39e-4
			*Gad67* ^+^ vs *Pomc*-only, *q* = 2.03, *p* = 0.48
			*Vglut2/Gad67* ^+^ vs *Pomc*-only, *q* = 7.88, *p* = 3.74e-6
6*P*	Normal	Linear regression	Slopes: *F*_(3,11)_ = 20.84, *p* = 7.66e-5
	distribution		*Vglut2* ^+^:
			*F*_(1,28)_ = 33.87, *p* = 2.97e-6, *R* ^2^ = 0.83
			*Gad67* ^+^ (levels 2–5):
			*F*_(1,22)_ = 57.49, *p* = 1.42e-7, *R* ^2^ = 0.98
			*Pomc*-only:
			*F*_(1,28)_ = 23.72, *p* = 3.95e-5, *R* ^2^ = 0.92
			*Vglut2/Gad67* ^+^:
			*F*_(1,28)_ = 39.83, *p* = 7.95e-7, *R* ^2^ = 0.83

## Results

### A lineage trace of *Vglut2*-tdTomato neurons revealed that approximately half of all immunoreactive POMC neurons were colabeled with tdTomato

**Figure 1.**
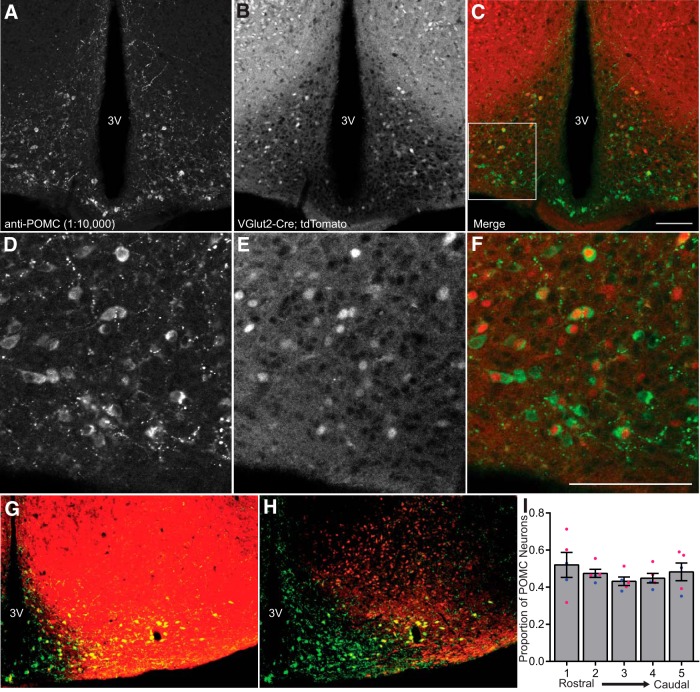
Genetic lineage trace of *Vglut2* expression and overlap with POMC IHC. ***A–C***, Confocal images of POMC immunoreactivity (green) from a male mouse (***A***), *Vglut2*-Cre-mediated tdTomato expression (red) (***B***), and overlap between the two signals (***C***). ***D–F***, 40× zoom of the inset outlined in panel ***C*** with the same signals as in ***A–C***. ***G–H***, 20× epifluorescent image from a female mouse with *post hoc* processing to identify tdTomato^+^-POMC overlap (yellow) in the medial (***G***) and lateral (***H***) ARC. ***I***, Quantification of the proportion of tdTomato^+^-POMC overlap throughout the rostral-caudal ARC axis. Male data for each group represented by filled blue circles and female data shown by filled pink circles. The scale bars represent 100 µm. 3V, third ventricle.

Because of differing reports for the percentage overlap of *Vglut2* expression in POMC neurons (Hentges et al., 2009; Vong et al., 2011; Dicken et al., 2012; Wittmann et al., 2013; Dennison et al., 2016), we initially performed a lineage trace by crossing *Vglut2*-IRES-Cre mice with floxed tdTomato mice. In contrast to data obtained by the laboratory that originally generated the *Vglut*2-IRES-Cre strain, which showed only a 10% overlap of their lineage trace with POMC neurons (Vong et al., 2011), we found that there was a nearly even split in the number of POMC neurons that were co-labeled or unlabeled with the *Vglut2*-tdTomato reporter (Fig. 1*A–H*). There was no effect of the rostral-caudal position in the ARC on the overlap counts (Fig. 1*I*). The reason for this large discrepancy in the fraction of glutamatergic POMC neurons is unclear, but there were technical differences in the identification method for POMC neurons. The previous group performed their lineage trace using a second transgenic reporter strain, POMC-hrGFP, and then quantified the coexpression of tdTomato and the surrogate marker hrGFP. We chose to quantify the overlap of tdTomato with POMC neurons using the immunohistochemical detection of POMC itself.

### Vglut2-Cre-mediated activation of *Pomc* expression in the hypothalamus of restored mice normalized body weight and composition, and POMC immunoreactivity

**Figure 2.**
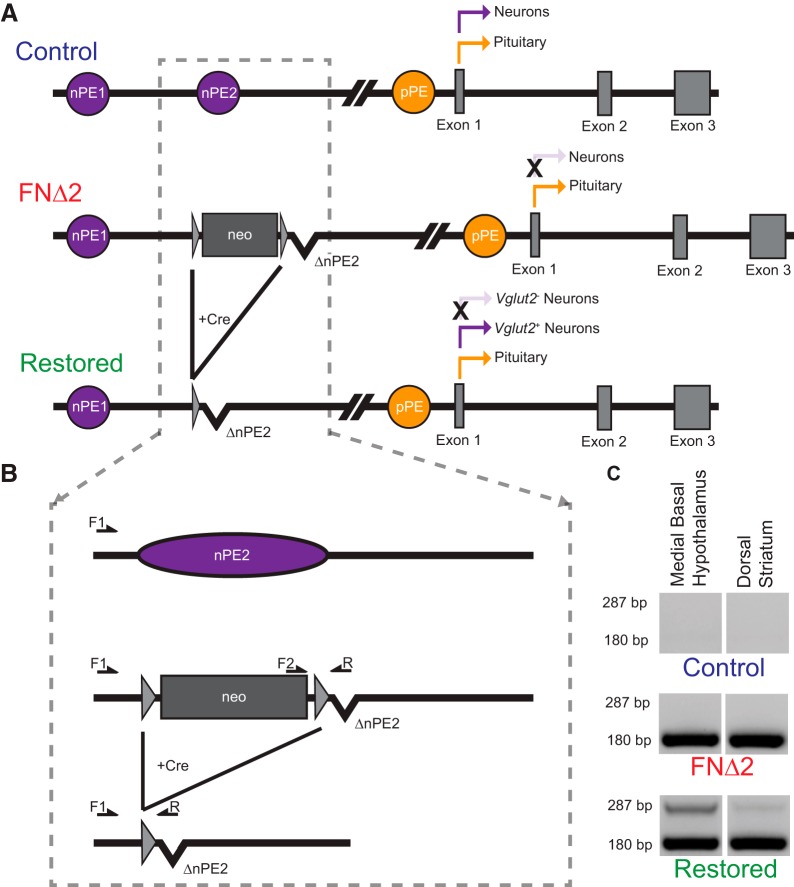
Vglut2-ires-Cre-specific recombination of the *Pomc* FNΔ2 allele. ***A***, Schematic representing the WT *Pomc* locus (control), the presence of the Cre-excisable floxed neomycin cassette along with the knock-out of nPE2 (FNΔ2), and the FNΔ2 allele after Cre-mediated excision leaving only the knock-out of nPE2 (restored). ***B*,** Hybridization location of the three oligonucleotide primer set used to assess the integrity of the Cre-mediated recombination. The forward 1 (F1) primer hybridizes upstream of nPE2, the forward 2 (F2) primer hybridizes to the intact neomycin cassette, and the reverse (R) primer is specific to the knocked out nPE2 locus. ***C*,** Verification of Cre-mediated genetic excision of the floxed-neomycin cassette from the *Pomc* neural enhancer locus in restored mice. The intensity of the recombined band (PCR product of F1 and R; 287 bp) versus the non-recombined band (PCR product of F2 and R; 180 bp) was much stronger in the medial basal hypothalamus than in the dorsal striatum. (*P* < 0.00001, see Table 2).

The specificity of *Vglut2*-ires-Cre mediated DNA recombination was assessed using PCR analysis of genomic DNA extracted from the relatively glutamatergic neuron-rich medial-basal hypothalamus and the relatively GABAergic neuron-rich dorsal striatum from restored mice. The three primer PCR design amplifies a 180-bp band from the intact FNΔ2 allele and a 287-bp band from the same allele after Cre-mediated recombination (Fig. 2*A*,*B*). No PCR product is generated from control DNA because the reverse primer cannot hybridize to the intact nPE2 enhancer. The degree of recombination was significantly higher in hypothalamic samples compared to striatal samples and there was no evidence of recombination in the brains of FNΔ2 mice lacking the *Vglut2*-IRES-Cre allele (Fig. 2*C*).

**Figure 3.**
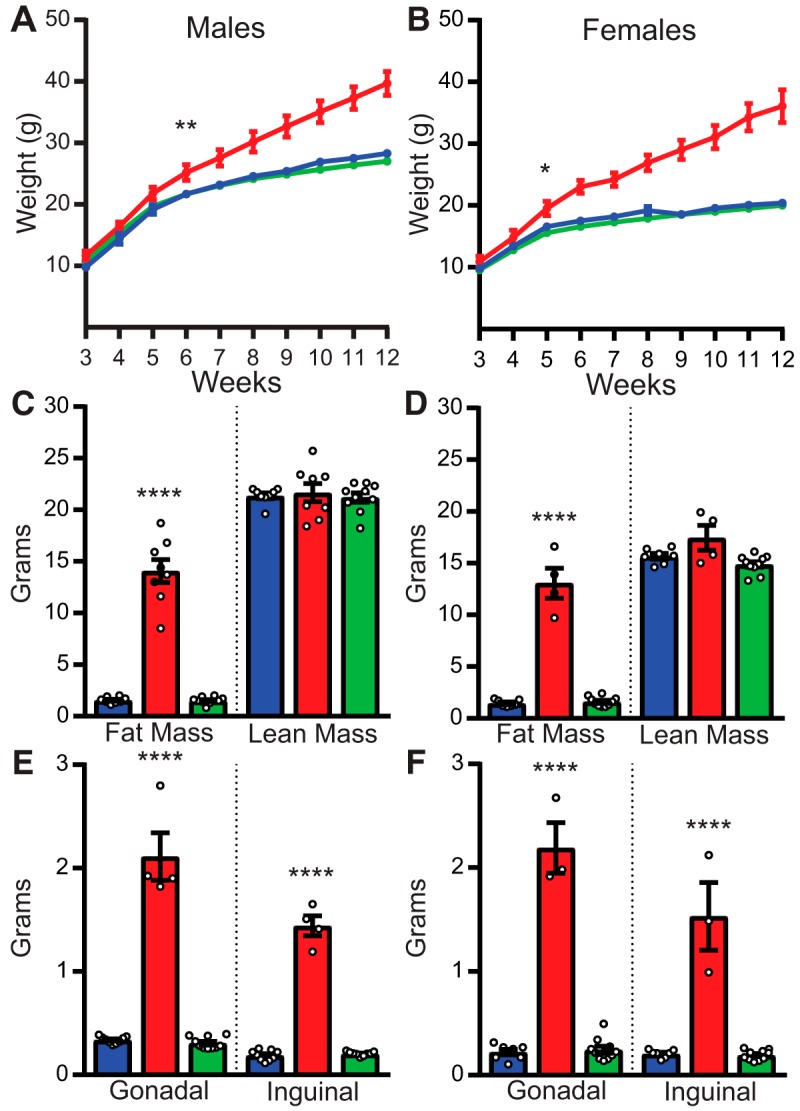
Vglut2-ires-Cre-mediated recombination of *Pomc* normalizes body composition. ***A*,** Growth curves from male mice. FNΔ2 (red line) mice significantly diverged from both control (blue line) and restored (green line) mice by six weeks of age. ***B***, Growth curves from female mice. FNΔ2 mice significantly diverged from both control and restored mice by five weeks of age. ***C***, ***D***, NMR assessment of body composition. Male (***C***) and female (***D***) FNΔ2 mice (red bars) had substantially more body fat than control (blue bars) or restored (green bars) mice, while there were no differences in lean mass. ***E***, ***F***, Weight of gonadal and inguinal fat depots. Male (***E***) and female (***F***) FNΔ2 mice (red bars) had substantially larger fat depots (both gonadal and inguinal) than control (blue bars) or restored (green bars) mice. *p < 0.05, **p < 0.01, ****p < 0.001.

Weekly body weights showed identical growth curves between restored and control mice. Both groups diverged significantly from FNΔ2 mice by age five (female) or six (male) weeks (Fig. 3*A*,*B*; only pairwise comparisons from one week prior to divergence to one week following are included in Table 2). Body composition measurements by NMR confirmed that these differences in body weight were due to excess fat mass in FNΔ2 mice compared to control and restored mice, with no differences in lean mass across all groups (Fig. 3*C*,*D*). Furthermore, weights of the gonadal and inguinal fat pads showed that there were no depot-specific differences between restored and control animals, while both fat pads in each group were substantially smaller than those from obese FNΔ2 mice (Fig. 3*E*,*F*).

**Figure 4.**
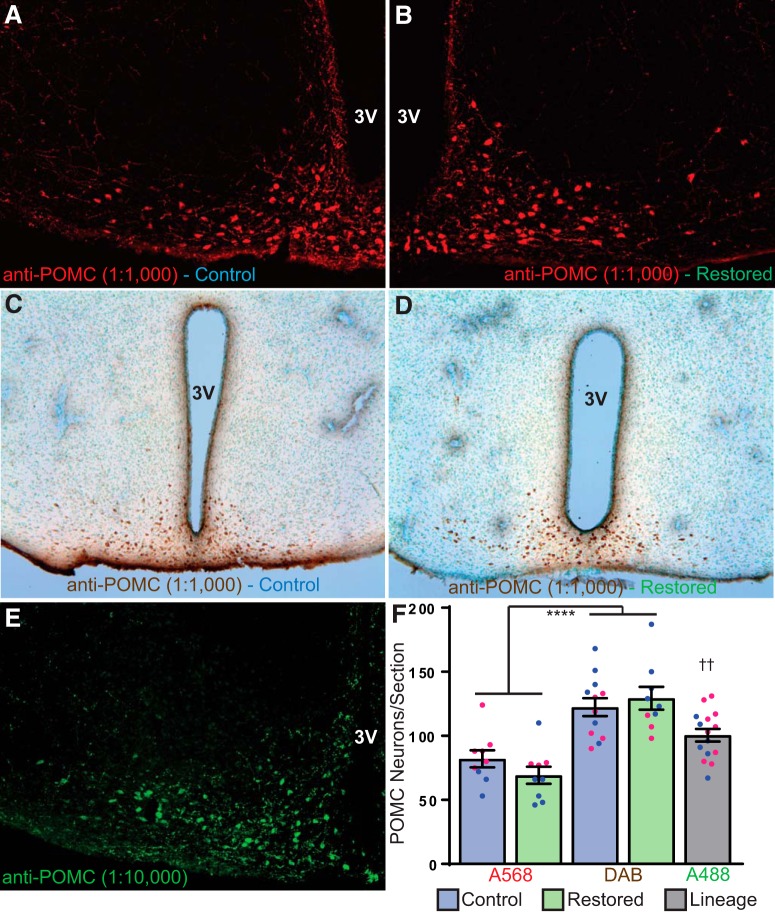
IHC for POMC cell counts in control and restored mice, and from VGlut2-Cre; tdTomato animals. ***A***, POMC-IR in a male control mouse detected with an Alexa Fluor 568 (red) secondary antibody (1:500). ***B***, POMC-IR in a male restored mouse detected with an Alexa Fluor 568 (red) secondary antibody (1:500). ***C***, POMC-IR in a female control mouse detected with biotinylated secondary antibody (1:500) and visualized with a diaminobenzidine (DAB) reaction (brown). ***D***, POMC-IR in a female restored mouse detected with biotinylated secondary antibody (1:500) and visualized with a DAB reaction (brown). ***E***, POMC-IR in a female Vglut2-Cre; tdTomato mouse detected with an Alexa Fluor 488 (green) secondary antibody (1:500; mirrored section from Fig. 1*G*,*H*). ***F*,** POMC neuron cell counts from sections (three per mouse). There was no difference between control (blue bars) or restored (green bars) mice, but only in the method of secondary labeling used. Male data for each group represented by filled blue circles and female data shown by filled pink circles; ****p < 1e-7, denotes the difference between all A568 counts compared to all DAB counts, ††*p* < 0.01, denotes the difference between all A488 counts compared to all A568 and DAB cell counts. 3V, third ventricle.

IHC for POMC was performed on neural tissue from control and restored mice. Three sections from each mouse taken between A-P coordinates from –1.5 to –1.9 mm posterior to bregma were included for analysis. There was no difference in either fluorescent (Fig. 4*A*,*B*) or DAB treated (Fig. 4*C*,*D*) cell counts between restored and control mice. However, there was an overall difference between labeling methods. DAB-treated sections displayed significantly higher cell counts than fluorescently-labeled sections (Fig. 4*E*).

### Dual-label ISH for *Pomc* and *Gad67* in restored mice identified a substantial population of *Gad67* ^+^ *Pomc* neurons

**Figure 5.**
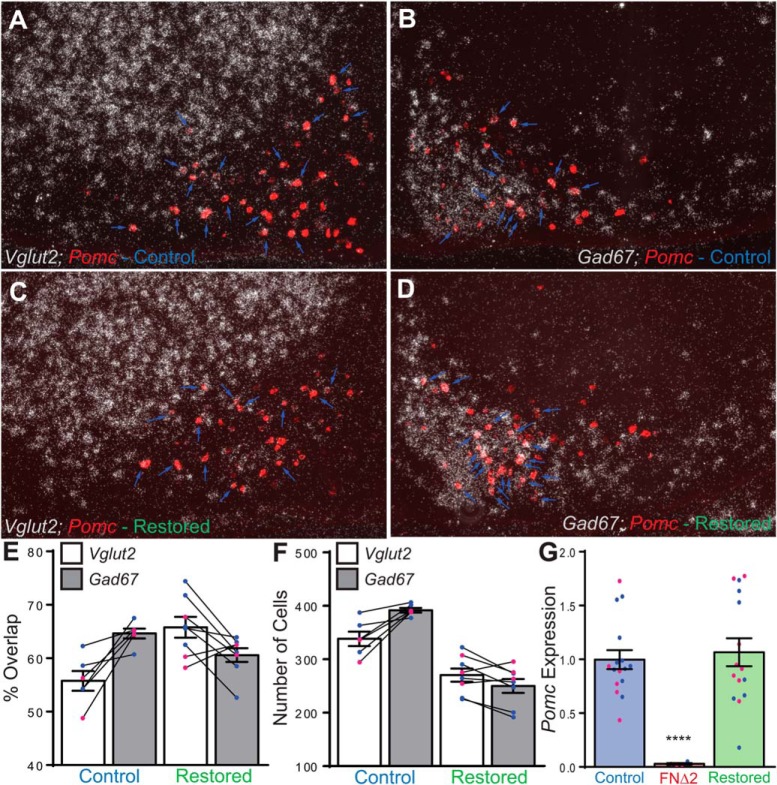
Dual-label ISH for *Pomc* and *Vglut2* or *Gad67*, and relative *Pomc* expression in the medial basal hypothalamus of control and restored mice. ***A***, ISH for *Vglut2* (silver grains) and *Pomc* (red) in a female control mouse. Note that the green fluorescence of Alexa Fluor 488 used to detect *Pomc* was pseudocolored to red for these images. ***B***, ISH for *Gad67* (silver grains) and *Pomc* (red) in a female control mouse. ***C***, ISH for *Vglut2* (silver grains) and *Pomc* (red) in a female restored mouse. ***D***, ISH for *Gad67* (silver grains) and *Pomc* (red) in a female restored mouse. In panels ***A–D***, blue arrows indicate overlap between *Pomc* and the silver grain (*Vglut2* or *Gad67*) signal. ***E***, Degree of overlap between *Pomc* and *Vglut2* (white bars) or *Gad67* (grey bars) in control and restored mice, each animal’s *Vlgut2/Pomc* and *Gad67/Pomc* overlap percentage is connected by the solid black lines. ***F***, Cell count of overlap between *Pomc* and *Vglut2* (white bars) or *Gad67* (grey bars) in control and restored mice, each animal’s *Vlgut2/Pomc* and *Gad67/Pomc* overlap count is connected by the solid black lines. ***G***, Relative qRT-PCR of *Pomc* expression in the medial-basal hypothalamus of control (blue bar, left), FNΔ2 (red bar, middle), and restored (green bar, right) mice. Male data are represented by filled blue circles and female data by filled pink circles.

While we did not measure any difference between control and restored mice with POMC IHC, we found significantly fewer total *Pomc* ^+^ cells in restored animals compared to controls using ISH (data not shown; Tables 1, 2; Fig. 5). Dual-label ISH showed that restored mice had a greater degree of *Pomc-Vglut2* and less *Pomc-Gad67* overlap than was observed in control animals (Fig. 5*A–E*). However, as noted earlier, the total number of *Pomc***-**labeled neurons was less in the restored animals, thus it follows that restored mice had less total *Pomc-Vglut2* and *Pomc-Gad67***-**labeled neurons than control animals (Fig. 5*F*). There was no difference in the cumulative sum of the percentage of *Pomc-Vglut2* and *Pomc-Gad67* cells between restored and control animals, indicating that each group possesses a proportionately comparable population of dual *Vglut2^+^/Gad67^+^* phenotype *Pomc* neurons. qRT-PCR for relative *Pomc* expression was performed on cDNA derived from RNA extracted from the medial-basal hypothalamus. Despite detecting fewer *Pomc* neurons with ISH, there was no difference in total steady-state *Pomc* mRNA levels between restored and control animals, and levels in both groups were ∼25-fold greater than FNΔ2 mice (Fig. 5*G*).

### Triple-label ISH in WT mice showed distinct *Pomc*-only, *Gad67^+^*/*Pomc*, *Vglut2^+^*/*Pomc*, and *Gad67^+^/Vglut2^+^*/*Pomc* neuron subpopulations with varying anatomic distribution along the rostral-caudal ARC axis

**Figure 6.**
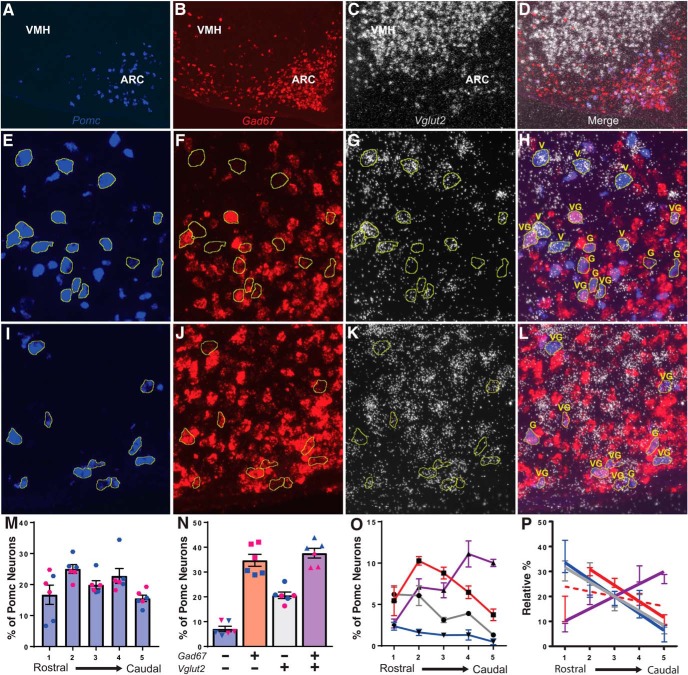
Triple-label ISH for *Pomc* (***A***, ***E***, ***I***), *Gad67* (***B***, ***F***, ***J***), *Vglut2* (C,G,K), and overlaid signals (***D***, ***H***, ***L***) in WT mice throughout the rostral-caudal ARC axis. ***A–D***, Low-magnification image of ISH signal for *Pomc*, *Gad67*, *Vglut2*, and the overlay of all signals from a male mouse. Note that the green fluorescence of Alexa Fluor 488 used to detect *Pomc* was pseudocolored to blue for these images. ***E–H***, 40× images from the rostral ARC from a male mouse with *Pomc* neuron profiles outlined in yellow. ***I–L***, 40× images from the caudal ARC from a female mouse with *Pomc* neuron profiles outlined in yellow. V indicates *Vglut2* ^+^ *Pomc* neurons, G indicates *Gad67* ^+^ *Pomc* neurons, and VG indicates *Vglut2/Gad67* ^+^ *Pomc* neurons. ***M***, The distribution of *Pomc* neurons along the rostral-caudal ARC axis. ***N***, The overall percentages of *Pomc*-only (blue bar with filled inverted triangles), *Gad67* ^+^ (red bar with filled squares), *Vglut2* ^+^ (grey bar with filled circles), and *Vglut2/Gad67* ^+^ (purple bar with filled triangles) *Pomc* neurons in the arcuate nucleus. Male data are represented by filled blue symbols and female data by filled pink symbols. ***O***, The percentages of *Pomc*-only (blue line with filled inverted triangles), *Gad67* ^+^ (red line with filled squares), *Vglut2* ^+^ (grey line with filled circles), and *Vglut2/Gad67* ^+^ (purple line with filled triangles) *Pomc* neurons at each coronal level 1 to 5 along the rostral-caudal ARC axis. ***P***, Linear regression analysis of the relative percentage of each phenotypic category of *Pomc* neurons along the rostral-caudal ARC axis [*Pomc*-only solid blue line, *Gad67* ^+^ dotted red line, *Gad67* ^+^ (levels 2–5) solid red line, *Vglut2* ^+^ solid grey line, *Vglut2/Gad67* ^+^ solid purple line].

The individual hybridization signals for *Pomc*, *Gad67*, and *Vglut2* from a representative hemi-section of the medial basal hypothalamus including the ARC and VMH are shown in Figure 6*A–C*. The merged image is shown in Figure 6*D*. Both *Pomc* and Gad67 expression are primarily located in the ARC while *Vglut2* mRNA is most densely located in the VMH. Neurons of interest were identified as shown in representative higher magnification sections from the rostral Arc (Fig. 6*E–H*) and the caudal ARC (Fig. 6*I–L*). There was a main effect on *Pomc* neuron counts along the rostral-caudal axis of the ARC. However, none of the Levels differed significantly from one another after *post hoc* multiple comparisons (Fig. 6*M*). Of the possible phenotypic combinations of *Pomc* expression overlapping with the two neurotransmitter markers throughout the entirety of the ARC, the *Pomc*-only cells constituted the smallest group, followed by double *Pomc/Vglut2^+^* and double *Pomc/Gad67* ^+^ neurons. Triple *Pomc/Vglut2^+^/Gad67* ^+^ cells comprised the largest group. Except for the *Pomc/Gad67* ^+^ count compared to the *Pomc/Vglut2/Gad67* ^+^ population, the size of every group was significantly different from every other group (Fig. 6*N*). Analysis of linear regression revealed several patterns that emerged along the rostral-caudal axis of the ARC. The *Pomc*-only and *Vglut2* ^+^ populations exhibited a similar trend of having the highest presence at the rostral end of the ARC and the lowest at the caudal end, while the *Vglut2/Gad67* ^+^ cells showed the opposite pattern. The *Gad67* ^+^ population did not exhibit a linear trend like the other groups, due to a sharp increase in expression between the two most rostral ARC positions. However, analyzing levels 2 through 5 showed that their numbers also diminished toward the caudal ARC (Fig. 6*O*,*P*).

We also observed a small number of dual *Vglut2/Gad67* ^+^ cells in the ARC that were not positive for *Pomc* mRNA. The phenotypic identity of these cells is not known. Furthermore, the density of non-*Pomc* dual *Vglut2/Gad67* ^+^ cells was higher in the DMN (data not shown). Together, these data indicate that neuron populations in the hypothalamus, other than POMC neurons, may also exhibit both glutamatergic and GABAergic characteristics.

## Discussion

The global impact of POMC peptides on metabolic homeostasis, body composition, and feeding behavior is widely recognized. However, studies investigating the fast synaptic transmission roles that POMC neurons serve are in their early stages. The initial rationale for conducting the current study was to decipher the physiological significance of glutamate-producing POMC neurons, using an experimental paradigm previously used to study leptin receptor POMC neurons (Lam et al., 2015a) and 5HT-2cR POMC neurons (Burke et al., 2016), by selective induction of *Pomc* transcription in these cell populations during hypothalamic development. We sought to characterize the function of a specific neuronal subset, but what we uncovered instead is a broader phenomenon of neural adaptation or plasticity.

The complete prevention of the obesity phenotype of FNΔ2 mice by *Vglut2*-ires-Cre induction of *Pomc* expression in hypothalamic neurons did not support our initial hypothesis that a subpopulation of glutamatergic POMC neurons would have selective effects on energy homeostasis. It is possible that more extensive metabolic phenotyping of the restored mice, including environmental challenges such as a high-fat diet, might have unveiled subtle alterations from control mice. However, the existing data for body weight growth over 12 weeks, normal body composition and normalized steady state *Pomc* mRNA levels at age 12 weeks, combined with the observed developmental alterations in neurotransmitter markers, suggested that additional metabolic phenotyping was unlikely to be informative or fully interpretable. Previous work indicated that *Pomc* mRNA levels above a threshold of 30% of control levels and evenly distributed spatially across the rostral-caudal axis of the ARC produced only a mild obesity phenotype in low fat chow fed mice (Lam et al., 2015b). Furthermore, *Pomc* mRNA levels above 50% of control levels protected mice from obesity, even when challenged with a high-fat diet (Lam et al., 2015b).

A first line of evidence for fast neurotransmitter plasticity within the POMC neuronal network arises from a discrepancy that we found between *Pomc* restoration in FNΔ2 mice and the *Vglut2* lineage trace experiments, even though each study was dependent on the same Cre-driver mouse strain. On one hand, we found a nearly complete restoration in the number of POMC-expressing neurons, indicating that all of those neurons either contemporaneously expressed or were derived from cells that had expressed *Vglut2* at some time-point. On the other hand, we found that only half of POMC immunoreactive cells were also positive for the lineage trace, suggesting that all POMC neurons could not have expressed or be derived from cells that were *Vglut2* ^+^ at some point. We can only speculate about the exact mechanism underlying these differences, but there may be a combination of multiple processes including sensitivity issues in the methods of detection between the two studies. Furthermore, distinct floxed alleles may have different thresholds for recombination by the same Cre-driver strain (Song and Palmiter, 2018).

It is possible that the restored mice had differences in developmental timing en route to normalization of the POMC system, which were not evident in measurements of body mass and composition. In the *Vglut2* lineage trace, normal POMC function should be intact and allow POMC neurons to follow a normal developmental program, resulting in an even split between tdTomato^+^ and tdTomato^-^ POMC neurons. In contrast, the *Pomc*-restoration experiment used FNΔ2 mice that are incapable of transcribing *Pomc* in neurons, and arguably undergo an altered developmental program that differentially impacts the hypothalamic landscape and ultimately global metabolic function. This fact is evident in the phenotypic traits of the latter mice: morbid obesity, hyperphagia, hypolocomotion, insulin resistance and alterations in glucose tolerance and glycosuria (Bumaschny et al., 2012; Chhabra et al., 2016a,b). However, when *Pomc* expression was induced in these mice by the *Vglut2*-IRES-Cre driver, the animals were phenotypically normal and had the same proportion of dual-labeled *Pomc-Gad67^+^* cells and POMC immunoreactive cells as control animals. Both of these results were unanticipated, we expected to restore function to only a subset of POMC neurons and that relatively few of them would be *Gad67* ^+^. We used GAD67 instead of VGAT as a marker of GABAergic phenotype because the latter transporter is not expressed in POMC neurons (Stincic et al., 2018). Despite this, there is substantial electrophysiological evidence of synaptic GABA release from POMC neurons. Together, these data suggest one or both of the following: *Gad67*-expressing POMC neurons also express *Vglut2*, and/or there is turnover and overlap from a glutamatergic identity to a GABAergic identity in POMC neurons. Whether these phenomena represent the normal pattern of activity for this cell population, or whether they arose from a compensatory mechanism initiated from an atypical developmental program, remains to be determined.

The differences that we found in cell counts between ISH and IHC experiments, as well as the difference observed between the ISH studies and the qRT-PCR measures, may be due to differences in the sensitivities in the assays used. The ISH experiments on the restored mice were conducted on tissue that was collected from animals that were between nine and 13 weeks old, whereas the IHC studies and qRT-PCR were conducted on tissue that was collected from mice that were between 12 and 13 weeks old. While this age gap is not typically regarded as a critical developmental period, there may be changes occurring in the neurotransmitter landscape, which could account for the different cell counts that we measured. The means of ISH tissue preparation and probing method used to label cells can profoundly impact the sensitivity and interpretation of the assay, which may account for the varying proportions of glutamatergic POMC neurons that have been reported via ISH, let alone IHC. However, our results fit with the numbers reported previously using the same preparation and detection methods (Hentges et al., 2009; Vong et al., 2011; Dicken et al., 2012; Wittmann et al., 2013; Dennison et al., 2016). For IHC, we used three different secondary antibodies and colorimetric or fluorescent techniques to label POMC neurons. The inconsistencies in our data were not between experimental groups, but between the detection methods that were used. We found the greatest number of POMC neurons using colorimetric DAB as the labeling method, followed by the fluorescent markers Alexa Fluor 488 and Alexa Fluor 568, respectively. However, we also utilized two different dilutions of the POMC primary antibody, 1:1000 for the DAB and Alexa Fluor 568 sections and 1:10,000 for the Alexa Fluor 488 sections. However, given that we measured a greater number of POMC neurons with Alexa Fluor 488 than with Alexa Fluor 568, we conclude that differences that we see are more a reflection of detection subjectivity of the secondary antibodies than of the efficiency of the primary antibody used.

Overlap between GABAergic and glutamatergic cellular phenotypes and machineries is not a novel concept. Several studies have identified instances of these intersections in multiple systems, which can be influenced by cellular excitability, developmental timing, or environmental factors. For example, Kao et al. (2004) found that there is a subset of retinal bipolar cells in cats that use both glutamate and GABA, and express vesicular transporters for each molecule. Using subcellular fractionation and synaptosomal isolation, Zander et al. (2010) identified sizeable vesicle pools containing both VGLUT2 and VGAT. Furthermore, they also showed that VGAT immunoisolates transport glutamate, and that VGLUT activity enhances the uptake of GABA and monoamines. Several studies have demonstrated that following stimulation-induced hyperexcitability of granule cells in the rat dentate gyrus, their mossy fibers can transition from a glutamatergic to a GABAergic neurotransmission and that these cells possess a GABAergic phenotype during early postnatal periods, which is suppressed in mature cells (Walker et al., 2001; Gutiérrez, 2002, 2003, 2005; Gutiérrez et al., 2003). In 2005, Gómez-Lira et al. (2005) further showed that the presence of TrkB-mediated BDNF signaling can also lead to a similar glutamatergic-to-GABAergic transition in mature cultured rat granule cells, showing that this phenomenon is not limited to a critical developmental period or supraphysiological stimulation. Ottem et al. (2004) also demonstrated that nearly all neurons in the female rat anteroventral periventricular nucleus are both GABAergic and glutamatergic and that both their excitability and VGLUT2 and VGAT vesicular pools are regulated by estradiol and photoperiodic signals, highlighting another case of external regulation of phenotypic identity. In the rat medial nucleus of the trapezoid body Gillespie et al. (2005) found GABA and glutamate co-release in the developing auditory system, which may aid in synaptic refinement and formation of the tonotopic map. More recently, Root et al. (2014) demonstrated in rats and mice that neurons from the ventral tegmental area innervating the lateral habenula (LHb), possess markers for both GABAergic and glutamatergic transmission. Furthermore, optogenetic stimulation of these cells exerts both excitatory and inhibitory influences on LHb neurons. Finally, a review by Mestikawy et al. (2011) outlines the overlap of VGLUT1, VGLUT2, and VGLUT3 with other transmitter systems throughout the central nervous system, highlighting the versatility and pervasiveness of this transporter family, both in function and anatomy. Beyond sharing neurotransmitter phenotypes, work from the Spitzer lab has highlighted complete switches from one neurotransmitter identity to another (Spitzer, 2015, 2017). In 2013, Dulcis et al. (2013) found a switch between dopamine and somatostatin neurons in response to exposure to different photoperiod lengths in adult mice, which was independent of neurogenesis or cell death. Meng et al. (2018) went on to show that in the rat paraventricular nucleus of the hypothalamus this switch occurs exclusively in dopaminergic neurons that coexpress VGLUT2 and that the switch is contingent on elevated activity in those cells.

Furthermore, coexpression of *Vglut2* and *Gad67* in *Pomc* neurons has been reported in a single-cell RNA sequencing study (Lam et al., 2017), where they observed a 24% overlap between the markers. They also reported that 87% of Pomc neurons express *Gad67* and 50% express *Vglut2*. The overall *Vglut2* and *Gad67* counts, and the triple counts seem to mostly be in agreement with the numbers that we measured in our dual- and triple-label ISH experiments, where we report more dual-labeled cells (38%) and total *Vglut2* ^+^ cells (dual-label: 56%, triple-label: 58%), but 15–22% less total *Gad67* ^+^ cells (dual-label: 65%, triple-label: 72%). The directionality of the *Vglut2* and *Gad67* counts between the studies are in agreement; where, *Gad67* represents a larger proportion of the *Pomc* population than *Vglut2*. Additionally, we can infer from their reported proportions that 13% of the sequenced *Pomc* neurons were *Pomc*-only, 50% were *Gad67^+^*, 13% were *Vglut2^+^*, and 24% were *Gad67^+^/Vglut2^+^*; these counts are somewhat different than our respective measures of 7.1%, 34.7%, 20.6%, and 37.6%. These differing counts could arise from a few factors: they used younger mice in their study, and their dataset arises from only 163 cells, which might not fully cover the entire *Pomc*-neuron population. Three other scRNAseq profiles of hypothalamic neurons have also identified the presence of the same four subpopulations of POMC neurons in regard to their coexpression of the two fast synaptic neurotransmitters (Campbell et al., 2017; Chen et al., 2017; Romanov et al., 2017).

An important question that can be asked regarding the restored mice arises from the lack of temporal control in the *Vglut2*-Cre induced, cell specific *Pomc* transcription. Given the widespread expression and subsequent decrease in *Vglut2* expression from early postnatal development to maturation (Boulland et al., 2004; Dennison et al., 2016), VGLUT2 alone may not be the best indication of mature glutamatergic neurons. It is also possible that other differentiated neurons in the ARC may transiently express VGLUT2 during development. For the current experiments with the restored mice, we were limited to the available genetic mouse lines, and the best option was a constitutively active Vglut2-Cre driver line. Using an inducible-Cre model could potentially provide more specific insight for the role of glutamatergic POMC neurons in adult animals. However, those experiments would come with their own caveats and considerations. One such consideration is that without sufficiently early intervention the FNΔ2 animals exhibit juvenile-onset obesity. An early history of excess adiposity could by itself permanently shape the hypothalamic landscape and its plasticity.

In conclusion, our triple ISH data highlight a previously overlooked phenomenon that could not be measured with previous reports using dual ISH alone. There is spatial heterogeneity between phenotypically different POMC neurons based on their markers of fast synaptic neurotransmitters along the rostral-caudal axis of the ARC. Previous work (Williams et al., 2010) demonstrated that there is an anatomical difference between leptin- and insulin-induced responses in POMC neurons. Whereas most leptin-induced hyperpolarization of POMC neurons occurred in the rostral ARC, most insulin-induced depolarization of POMC neurons occurred in the caudal portion of the ARC. Furthermore, Betley et al. (2013) demonstrated in AgRP neurons that the rostral-caudal location of the cell bodies was associated with their projection targets, where the most rostral neurons tended to project to forebrain areas, while the caudally located cells projected to hindbrain areas. Together, these findings raise questions of how overall ARC function changes developmentally and whether other ARC neuron subtypes, such as kisspeptin and dopamine neurons, also exhibit phenotypic shifts in their fast synaptic neurotransmitters.

## Synthesis

Reviewing Editor: Margaret McCarthy, Univ of Maryland School of Medicine Decisions are customarily a result of the Reviewing Editor and the peer reviewers coming together and discussing their recommendations until a consensus is reached. When revisions are invited, a fact-based synthesis statement explaining their decision and outlining what is needed to prepare a revision will be listed below. The following reviewer(s) agreed to reveal their identity: Denise Belsham, Holly Ingraham.

Two expert reviewers examined the manuscript and found much merit but also expressed concern about over interpretation of some of the data which was viewed as preliminary. I have divided their comments into positive and negative, followed by a numbered list of items that need to be attended to.

Generalized positive comments

This manuscript opens a new line of investigation with robust experimental design and novel results. The question of neuronal plasticity is moving into the forefront and perhaps dogmas will fall, in terms of the classic idea of specific markers in defined neurons over their lifespan. This is worth pursuing, particularly how this impacts the control of energy homeostasis.

This study aims to determine the contribution of glutamate to the overall function of POMC neurons utilizing sophisticated mouse models that restore POMC expression specifically in neurons expressing Vglut2. Further the authors study whether there are subpopulations of POMC neurons that co-express glutamate and GABA. Restoring POMC only in glutamatergic neurons rescued the effect of POMC deletion on body composition, specifically fat mass. Interestingly, the paper then focusses on the delineation of Gad67- and Vglut2-specific neurons, and the potential plasticity in expression of these enzymes in POMC neurons. While the overall number of POMC neurons does not change rostral to caudal, it appears that the number of POMC neurons co-expressing Gad67 and Vglut2 is increasing caudally. The reason for this plasticity is not clear. In general, this is an impressive manuscript, particularly with the level of technical difficulty that the study represents. The use of triple labeling is somewhat rare to see in brain studies and the information provided is believable and robust.

Generalized concerns

Overall this study is of potential interest, but as currently presented there are 3 independent and descriptive stories, none of which offers a clear and concise view of how their data fits into an overall theme of POMCARC neuronal development or function. The major finding of this study shows that restoring POMC to excitatory ARC neurons fully restores obesity phenotype. This is an important observation and provides an excellent starting point to fully explore the functional role of VGlut2 and GABA POMC ARC neurons. In this sense this study would be appropriate for eNeuro. The other conclusions (regional differences and developmental flexibility) are less developed with current data and are far less solid and more speculative. Other methods to obtain better data to visualize overlap of Vglut2 and POMC cells would strengthen their case (RNA-Scope, fluorescein-labeled cRNA, View-RNA) and functional outputs would also help.

The main question posed by this paper - what is the role of the fast transmission in POMC neurons - is not answered by the results from this work. Defining the physiological role of Vglut2 neurons is still not clear, as it appears that there may be an increased level of Gad67/Vglut2 co-expression in the restored POMC neurons, confounding the situation even more. So what is the purpose of this neural adaptation or plasticity? Is it just a phenomenon produced by the use of transgenics or does this occur naturally? This is still unanswered.

Specific comments:

1. The differences in sensitivity between IHC detection methods were quite interesting. The authors indicate that there was a difference between Alexa Fluor 488 and Alexa Fluor 568, and was discussed. Both should be included in Fig 4E.

2. It is not clear whether the representative images included in the figures were taken from male or female mice, please include this information in the figure legends. From the data in Fig 1I, it appears that only two males were used per group, which is not sufficient to compare differences in male to female mice. The number of experimental replicates (with each sex) should be included in the figure legends. Need to break out data on males and females in all figures to determine if there are some interesting sex-differences, especially given that ERa is expressed in POMCARC neurons.

3. It would be interesting to know if ERa is expressed in their three different subtypes of POMCARC neurons.

4. Were the mice sacrificed at the same time of day? Perhaps the difference in expression levels of the Gad67 and Vglut2 is dependent on time of day (circadian regulation)?

5. The authors present a balanced, interesting discussion. For completeness, I would appreciate a short description of the relevance of the rostral versus caudal Arc, if anything is known in this area. What might be the significance of an increase in double-labeled neurons in the caudal region?

6. Further, it is somewhat surprising that the authors did not cite nor discuss the recent paper by Lam...Yeo (Mole Metab 2017) that reports on the heterogeneity of POMC neurons using single cell RNA-seq. This paper actually reported that 24% of POMC neurons express both Gad67 and Vglut2, as well as the individual expression levels in Arc POMC neurons. Certainly this manuscript must be discussed and assessed for similarities and differences.

7. So the overall question remains - is this an experimentally-induced phenomenon or an important brain function to respond to stimulation or insult? This is unclear, however, this does not diminish the importance and interest in the present study. The paper opens a new line of investigation with robust experimental design and novel results. The question of neuronal plasticity is moving into the forefront and perhaps dogmas will fall, in terms of the classic idea of specific markers in defined neurons over their lifespan. This is worth pursuing, particularly how this impacts the control of energy homeostasis.

## Author Response

February 14, 2019

Editor

eNeuro

Dear Dr. McCarthy,

We thank you and the reviewers for the time and consideration that went into reviewing our manuscript and preparing the feedback. We are glad that it was for the most part well received and hope that after addressing the concerns raised, our paper will aid in shaping future studies on the neuroplasticity of fast neurotransmitter synthesis and the functional role of GABA and glutamate release from subpopulations of POMC neurons.

To comment on the general concerns, we agree that the stories included are slightly discontinuous, and that we could only speculate on how they fully transition one to another. The discussion section has been updated to better place our findings in the overall understanding of the ARC POMC landscape. We didn't intend to over interpret any of the findings, but to just present possibilities that we have discussed, and the questions we have asked, which may help explain our results and provide parsimonious explanations for the findings of all of the studies together. We agree that future studies might benefit from the cellular resolution and quantitative possibilities of newer ISH techniques, but they have not yet been fully developed and validated in our labs.

Responses to specific comments:

1. The differences in sensitivity between IHC detection methods were quite interesting. The authors indicate that there was a difference between Alexa Fluor 488 and Alexa Fluor 568, and was discussed. Both should be included in Fig 4E.

Figure 4 and the corresponding statistics have been updated to include the Alexa Fluor 488 data, which was measured in the Vglut2-tdTomato lineage trace experiment presented in Figure 1.

2. It is not clear whether the representative images included in the figures were taken from male or female mice, please include this information in the figure legends. From the data in Fig 1I, it appears that only two males were used per group, which is not sufficient to compare differences in male to female mice. The number of experimental replicates (with each sex) should be included in the figure legends. Need to break out data on males and females in all figures to determine if there are some interesting sex-differences, especially given that ERa is expressed in POMCARC neurons.

We have updated the figure legends, annotating the sex of the animals used for the representative images; the replicate information is in Table 1. We also updated Figure 5E, 5F, 6M, and 6N to represent the female-male identities of the data points. Overall, there is no suggestion of relevant sex-differences.

3. It would be interesting to know if ERα is expressed in their three different subtypes of POMCARC neurons.

We cannot attribute an ERα identity to any of the specific cells characterized in our studies. However, we did acquire and analyze all of the Pomc+ single cell RNA-sequencing data sets available from mice under basal conditions (Lam et al. 2017 Molecular Metabolism, Romanov et al. 2017 Nature Neuroscience, Campbell et al. 2017 Nature Neuroscience, and Chen et al. 2017 Cell Reports. There were a total of 1,358 Pomc+ cells from the combined data sets, of which the following percentages were also positive for Esr1 expression: 12.9% Pomc-only, 19.2% Gad67+/Pomc-positive, 29.7% Vglut2+/Pomc-positive, and 40.1% Gad67+/Vglut2+ /Pomc-positive. Therefore, ERα is expressed in all four POMC neuronal subtypes that we identified.

4. Were the mice sacrificed at the same time of day? Perhaps the difference in expression levels of the Gad67 and Vglut2 is dependent on time of day (circadian regulation)?

Tissue used for the dual label ISH was collected on two different dates, where we tried to keep the time of sacrifice as close as possible in the late morning, and we didn't observe any differences in cell counts between the dates of collection. Tissue used for the triple label ISH was collected all at once within an hour of each other in the middle of the light cycle. The cumulative totals of all Vglut2+- and Gad67+-Pomc neurons were similar between the studies.

5. The authors present a balanced, interesting discussion. For completeness, I would appreciate a short description of the relevance of the rostral versus caudal Arc, if anything is known in this area. What might be the significance of an increase in double-labeled neurons in the caudal region?

Great suggestion, we have included additional discussion on the topic of rostral-caudal differences in ARC POMC neurons.

6. Further, it is somewhat surprising that the authors did not cite nor discuss the recent paper by Lam...Yeo (Mole Metab 2017) that reports on the heterogeneity of POMC neurons using single cell RNA-seq. This paper actually reported that 24% of POMC neurons express both Gad67 and Vglut2, as well as the individual expression levels in Arc POMC neurons. Certainly this manuscript must be discussed and assessed for similarities and differences.

This is another great point, and we apologize for our oversight of this important study. We have included comparison to Lam et al's report of scRNAseq from POMC neurons in the text, as well as incorporating their dataset into our Esr1 screen as described above in comment #3.

7. So the overall question remains - is this an experimentally-induced phenomenon or an important brain function to respond to stimulation or insult? This is unclear, however, this does not diminish the importance and interest in the present study. The paper opens a new line of investigation with robust experimental design and novel results. The question of neuronal plasticity is moving into the forefront and perhaps dogmas will fall, in terms of the classic idea of specific markers in defined neurons over their lifespan. This is worth pursuing, particularly how this impacts the control of energy homeostasis.

We would love to have an answer to this question, but right now we cannot shed any more light beyond the potential of neurotransmitter flexibility. However, we do believe that our findings warrant studying this phenomenon under more naturalistic conditions, such as transition from limited resource availability to excess, drastic shifts in diet composition between high- and low-fat, or changes in photoperiod length, all experiments that are beyond the scope of this report.

Finally, if our revised manuscript is accepted for publication, the statement in the methods section concerning IACUC status, ‘All animal procedures were performed in accordance with the [Author University] animal care committee's regulations’ should be replaced with, ‘All animal procedures were approved by and performed in accordance with the University of Michigan's IACUC regulations.’

Sincerely,

Malcolm J. Low, MD, PhD

David F. Bohr Collegiate Professor of Physiology
